# Supplementary material for: A Phase II trial of alternating osimertinib and gefitinib therapy in advanced EGFR-T790M positive non-small cell lung cancer: OSCILLATE
Source: Nat Commun. 2024 Feb 28;15:1823. doi: 10.1038/s41467-024-46008-1 (PMC10902357; doi:10.1038/s41467-024-46008-1)
Supplement: Supplementary file 1 — Supplementary Information [file 41467_2024_46008_MOESM1_ESM.pdf]

A Phase II trial of alternating osimertinib and gefitinib in advanced *EGFR*-T790M positive non-small cell lung cancer: OSCILLATE

Lavinia Tan, Chris Brown, Antony Mersiades, Chee Khoon Lee, Thomas John, Steven Kao, Genni Newnham, Kenneth O'Byrne, Sagun Parakh, Victoria Bray, Kevin Jasas, Sonia Yip Stephen Q. Wong, Sarah Ftouni, Jerick Guinto, Sushma Chandrashekar, Stephen Clarke, Nick Pavlakis, Martin R. Stockler, Sarah-Jane Dawson, Benjamin J. Solomon

SUPPLEMENTARY APPENDIX

Supplementary Figure 1. Kaplan-Meier estimate of progression-free survival-2 (PFS2) for evaluable participants (n=30). 5

Supplementary Figure 2. Kaplan-Meier estimate of overall survival (OS) for evaluable participants (n=46) stratified according to activating EGFR mutation (EGFRm) type. 6

Supplementary Figure 3. Kaplan-Meier estimate of (a) PFS and (b) OS for participants (n=47) stratified according to sex. 8

Crosses denote censored observations, and each time interval the number of participants at risk are indicated below the plots. Comparisons were made using a two-sided log-rank test. PFS, progression-free survival;, OS, overall survival. 8

Supplementary Figure 4. Kaplan-Meier estimate of (a) PFS and (b) OS for participants (n=38) with a detectable *TP53* mutation versus *TP53* wild-type in baseline plasma. 10

Supplementary Figure 5. Kaplan-Meier estimate of (a) PFS and (b) OS for participants (n=38) with a detectable *EGFR* amplification versus no *EGFR* amplification in baseline plasma. 12

Supplementary Figure 6. Kaplan-Meier estimate of (a) PFS and (b) OS for participants (n=38) with a detectable *MET* amplification versus no *MET* amplification in baseline plasma. 14

Supplementary Figure 7. Co-alterations detected in baseline plasma in participants with T790M:EGFRm$_R$ ≤median and >median. 15

Supplementary Figure 8. Consort diagram of plasma samples analyzed from OSCILLATE trial. 17

Supplementary Figure 9. Dynamics of T790M mutant DNA copies/mL between baseline and week 4 (n=36), and baseline and week 12 (n=34) in participants achieving PR, SD, and PD. 19

Supplementary Figure 10. a) EGFRm W4-BLR and b) EGFRm W12-BLR for participants achieving PR, SD, or PD. 21

Supplementary Figure 11. a) T790M W4-BL_R and b) T790M W12-BL_R for participants achieving PR, SD, or PD.. 23

Supplementary Figure 12. Kaplan-Meier estimate of a) PFS and b) OS for participants (n=35 and n=36, respectively) stratified according to EGFRm W4-BL_R ≤median and >median. 25

Supplementary Figure 13. Kaplan-Meier estimate of a) PFS and b) OS for participants (n=35 and n=36, respectively) stratified according to T790M W4-BL_R ≤median and >median. 27

Supplementary Figure 14. Kaplan-Meier estimate of PFS for participants (n=31) stratified according to EGFRm W12-BL_R ≤median and >median. 28

Supplementary Figure 15. Kaplan-Meier estimate of PFS for participants (n=31) stratified according to T790M W12-BL_R ≤median and >median. 29

Supplementary Figure 16. Kaplan-Meier estimate of a) PFS and b) OS for participants (n=35 and n=36, respectively) stratified according to clearance versus non-clearance of EGFRm DNA (copies/mL) by week 4. 31

Supplementary Figure 17. Kaplan-Meier estimate of PFS for participants (n=31) stratified according to clearance versus non-clearance of EGFRm DNA (copies/mL) by week 12..... 32

Supplementary Figure 18. Kaplan-Meier estimate of a) PFS and b) OS for participants (n=33 and n=34, respectively) stratified according to clearance versus non-clearance of T790M DNA (copies/mL) by week 4. 34

Supplementary Figure 19. Kaplan-Meier estimate of PFS for participants (n=30) stratified according to clearance versus non-clearance of T790M mutant DNA (copies/mL) by week 12. 35

SUPPLEMENTARY TABLES

Supplementary Table 1. Concordance of baseline plasma *EGFR*m and T790M mutation detection between ddPCR and targeted sequencing assays……………………………….36

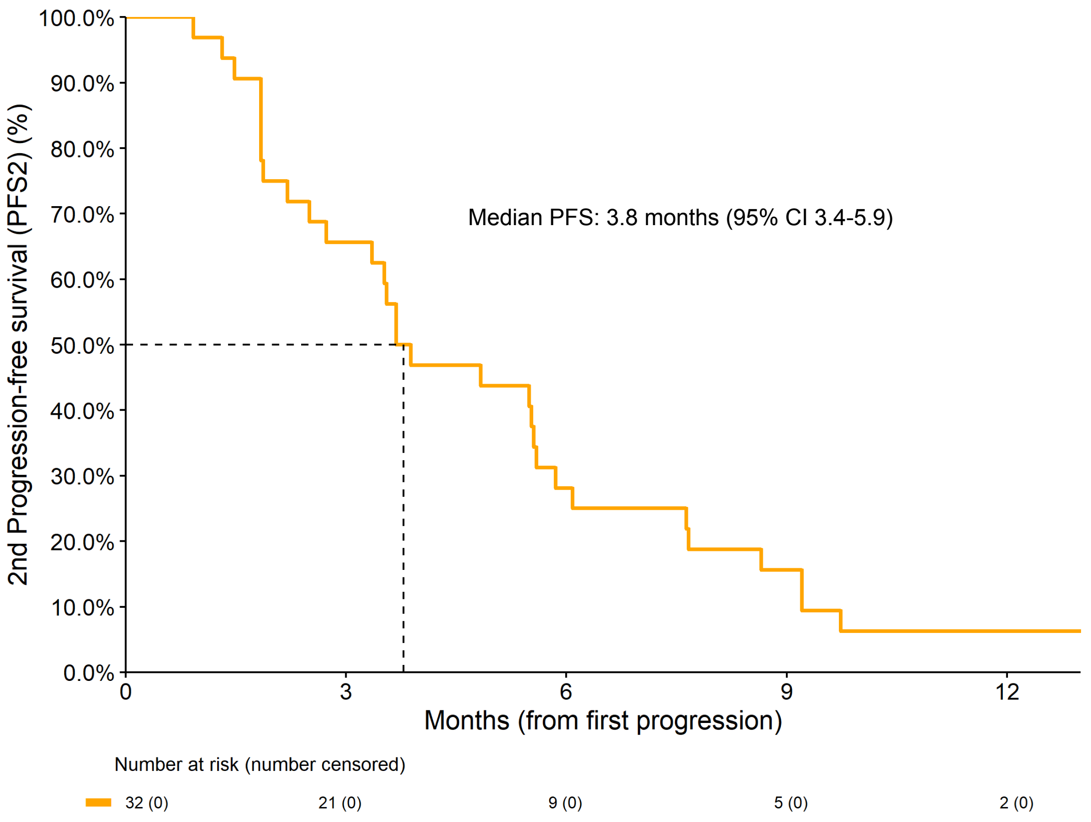

Supplementary Figure 1. Kaplan-Meier estimate of progression-free survival-2 (PFS2) for evaluable participants (n=30). Source data are provided as source data file**.**

Median PFS is shown as the horizontal and vertical dashed line. For each time interval, the number of participants at risk are indicated below the plots. 95% CI, 95% confidence interval.

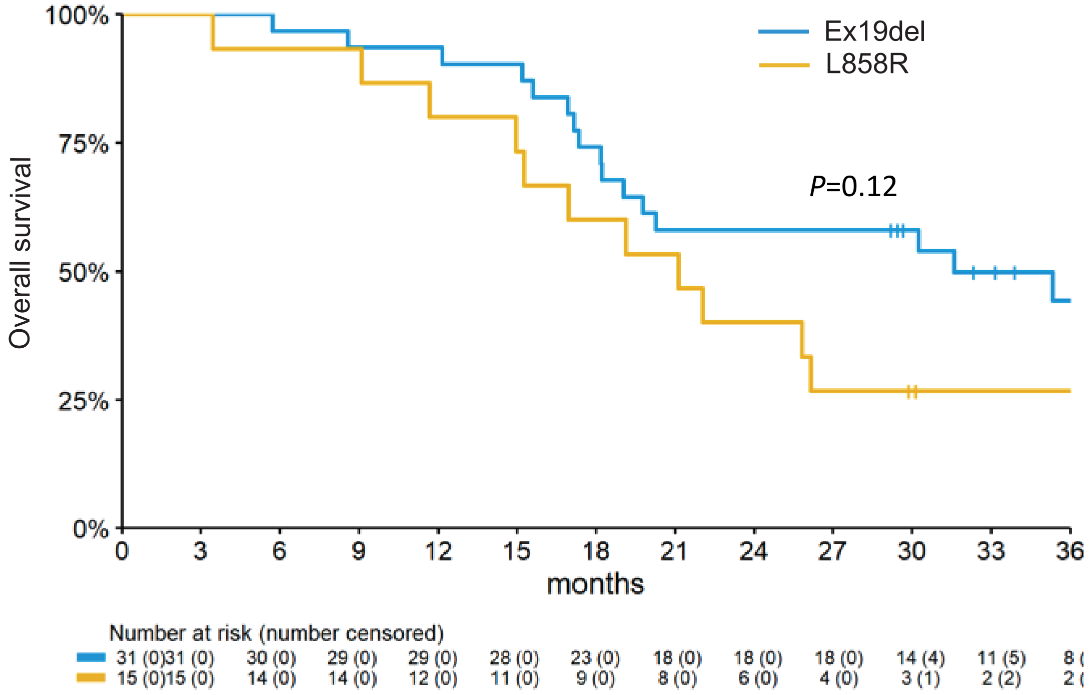

Supplementary Figure 2. Kaplan-Meier estimate of overall survival (OS) for evaluable participants (n=46) stratified according to activating EGFR mutation (EGFRm) type. Source data are provided as source data file**.**

Crosses denote censored observations, and the number of participants at risk are indicated below the plots at each time interval. Comparisons were made using a two-sided log-rank test. EGFRm, epidermal growth factor receptor mutation; Ex19del, exon 19 deletion.

a)

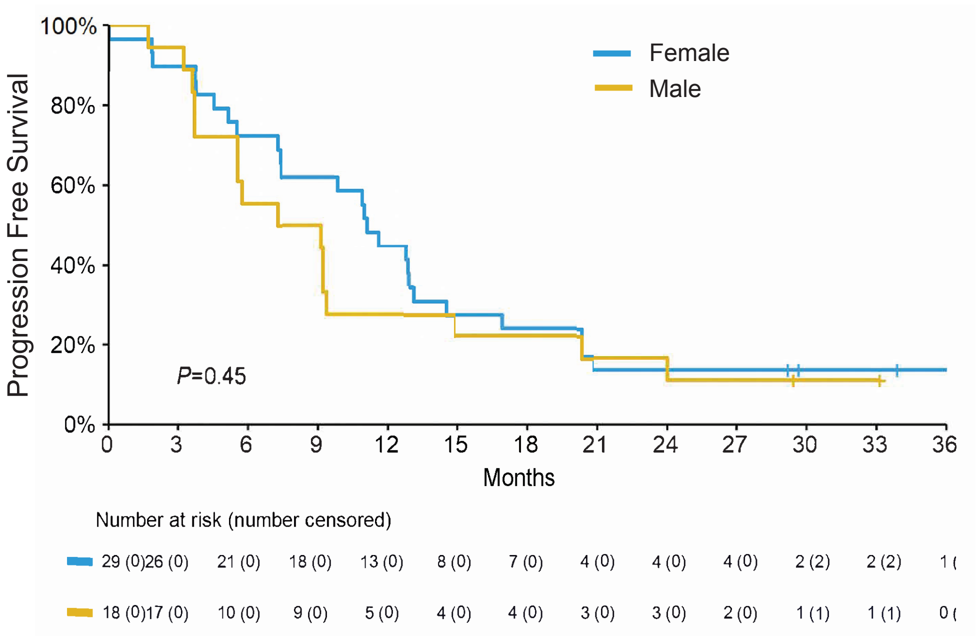
Female
Male
Progression Free Survival
100%
80%
60%
40%
20%
0%
P=0.45
0 3 6 9 12 15 18 21 24 27 30 33 36
Months
Number at risk (number censored)
29 (0)26 (0) 21 (0) 18 (0) 13 (0) 8 (0) 7 (0) 4 (0) 4 (0) 4 (0) 2 (2) 2 (2) 1 (
18 (0)17 (0) 10 (0) 9 (0) 5 (0) 4 (0) 4 (0) 3 (0) 3 (0) 2 (0) 1 (1) 1 (1) 0 (

b)

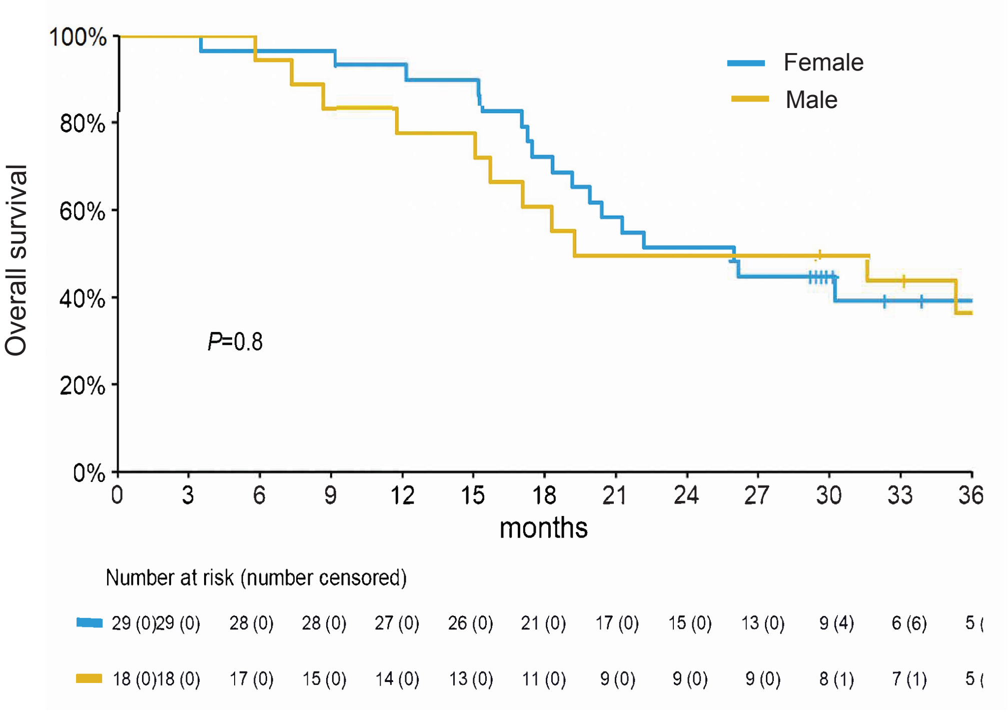
Female
Male
Overall survival
100%
80%
60%
40%
20%
0%
P=0.8
0 3 6 9 12 15 18 21 24 27 30 33 36
months
Number at risk (number censored)
29 (0)29 (0) 28 (0) 28 (0) 27 (0) 26 (0) 21 (0) 17 (0) 15 (0) 13 (0) 9 (4) 6 (6) 5 (
18 (0)18 (0) 17 (0) 15 (0) 14 (0) 13 (0) 11 (0) 9 (0) 9 (0) 9 (0) 8 (1) 7 (1) 5 (

Supplementary Figure 3. Kaplan-Meier estimate of (a) PFS and (b) OS for participants (n=47) stratified according to sex.

Crosses denote censored observations, and the number of participants at risk are indicated below the plots at each time interval. Comparisons were made using a two-sided log-rank test. PFS, progression-free survival; OS, overall survival.

a)

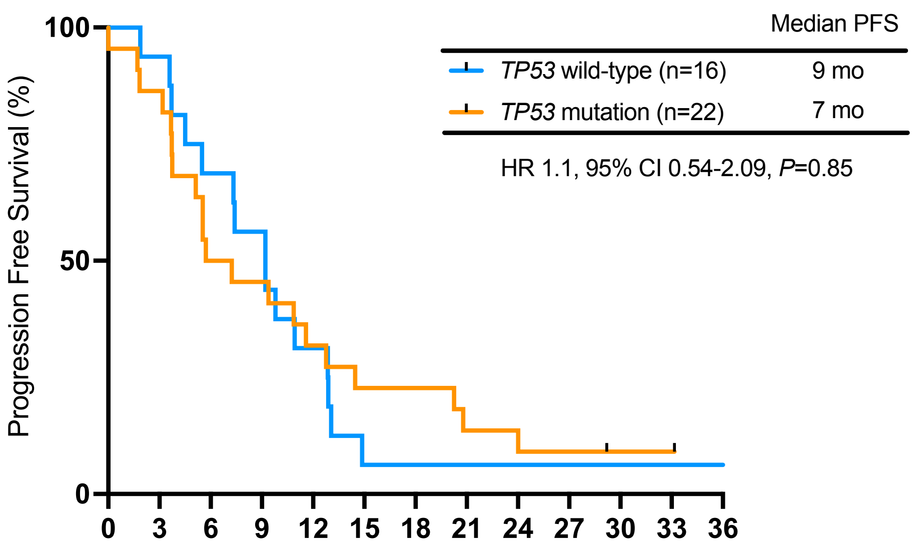

No. at risk:

TP53 wild-type	16	15	11	9	5	1	1	1	1	1	1	1	1
TP53 mutation	22	19	11	10	7	5	5	3	3	2	1	1	0

b)

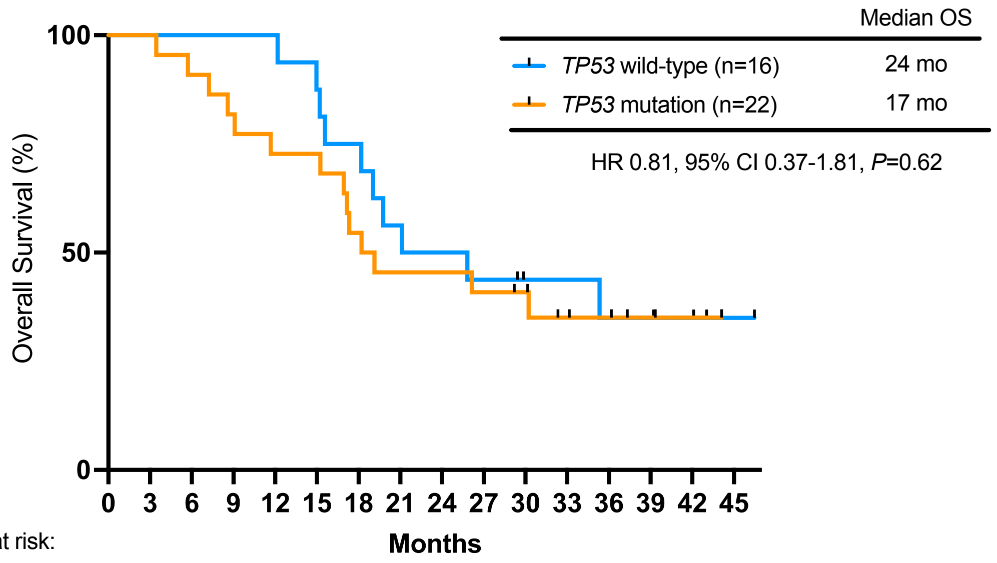

No. at risk:

TP53 wild-type	16	16	16	16	16	14	12	10	10	9	8	5	4	4	3	1
TP53 mutation	22	22	20	18	16	16	12	10	10	9	8	5	4	4	2	1

Supplementary Figure 4. Kaplan-Meier estimate of (a) PFS and (b) OS for participants (n=38) with a detectable *TP53* mutation versus *TP53* wild-type in baseline plasma.

Source data are provided as source data file**.**

Black vertical lines denote censored observations, and the number of participants at risk are indicated below the plots at each time interval. Comparisons were made using a two-sided log-rank test. *TP53*, tumour protein p53; PFS, progression-free survival; OS, overall survival; HR, hazard ratio; 95% CI, 95% confidence interval.

a)

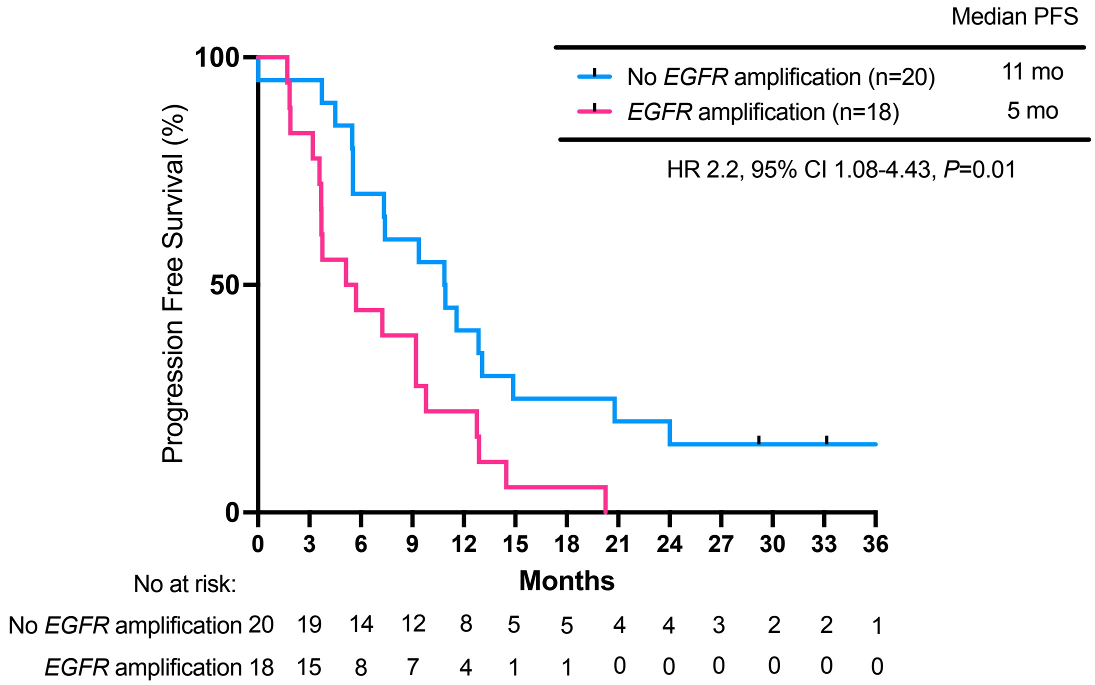
Median PFS
No EGFR amplification (n=20) 11 mo
EGFR amplification (n=18) 5 mo
HR 2.2, 95% CI 1.08-4.43, P=0.01
Progression Free Survival (%)
100
50
0
0 3 6 9 12 15 18 21 24 27 30 33 36
Months
No at risk:
No EGFR amplification 20 19 14 12 8 5 5 4 4 3 2 2 1
EGFR amplification 18 15 8 7 4 1 1 0 0 0 0 0 0

b)

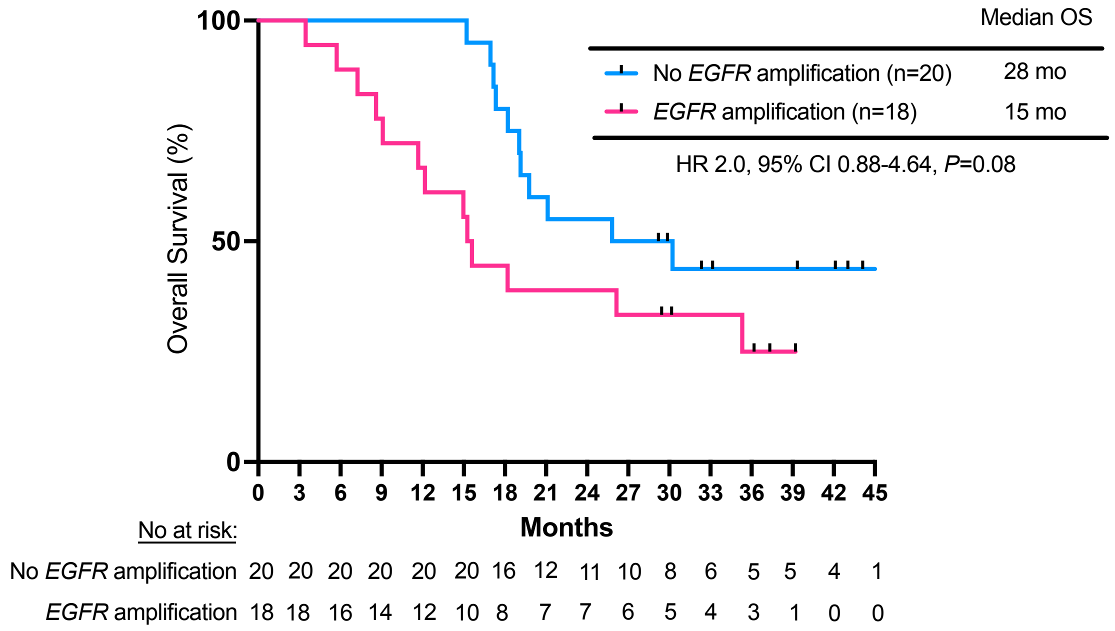
Median OS
No EGFR amplification (n=20) 28 mo
EGFR amplification (n=18) 15 mo
HR 2.0, 95% CI 0.88-4.64, P=0.08
Overall Survival (%)
100
50
0
0 3 6 9 12 15 18 21 24 27 30 33 36 39 42 45
Months
No at risk:
No EGFR amplification 20 20 20 20 20 20 16 12 11 10 8 6 5 5 4 1
EGFR amplification 18 18 16 14 12 10 8 7 7 6 5 4 3 1 0 0

Supplementary Figure 5. Kaplan-Meier estimate of (a) PFS and (b) OS for participants (n=38) with a detectable *EGFR* amplification versus no *EGFR* amplification in baseline plasma. Source data are provided as source data file**.**

Black vertical lines denote censored observations, and the number of participants at risk are indicated below the plots at each time interval. Comparisons were made using a two-sided log-rank test. *EGFR*, epidermal growth factor receptor; PFS, progression-free survival; OS, overall survival; HR, hazard ratio; 95% CI, 95% confidence interval.

a)

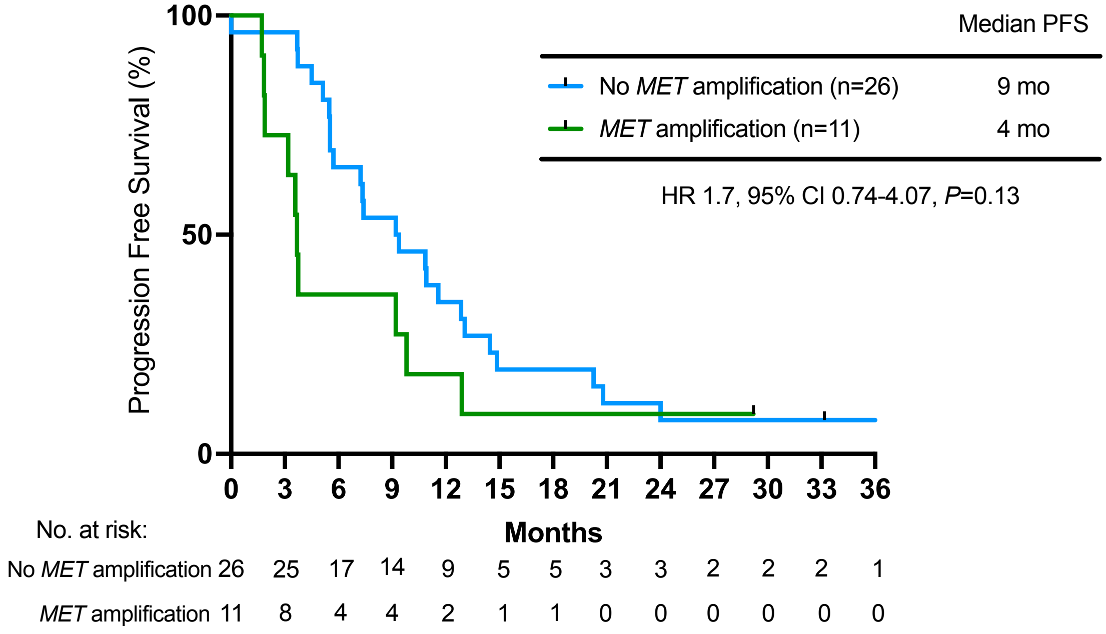
Median PFS
No MET amplification (n=26) 9 mo
MET amplification (n=11) 4 mo
HR 1.7, 95% CI 0.74-4.07, P=0.13
Progression Free Survival (%)
100
50
0
0 3 6 9 12 15 18 21 24 27 30 33 36
Months
No. at risk:
No MET amplification 26 25 17 14 9 5 5 3 3 2 2 2 1
MET amplification 11 8 4 4 2 1 1 0 0 0 0 0 0

b)

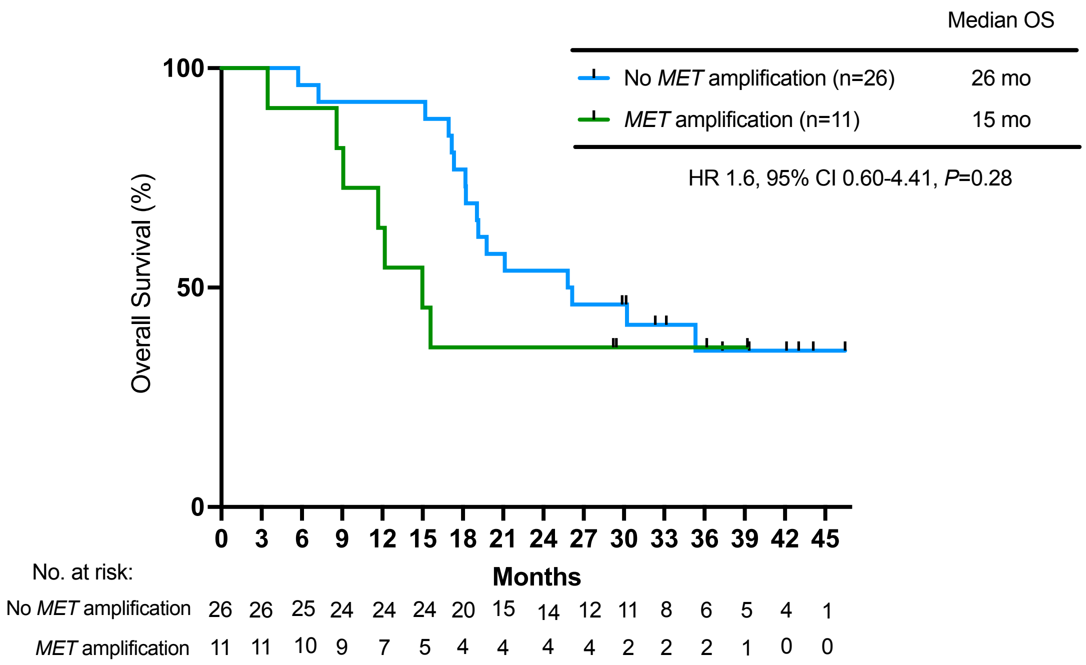
Median OS
No MET amplification (n=26) 26 mo
MET amplification (n=11) 15 mo
HR 1.6, 95% CI 0.60-4.41, P=0.28
Overall Survival (%)
100
50
0
0 3 6 9 12 15 18 21 24 27 30 33 36 39 42 45
Months
No. at risk:
No MET amplification 26 26 25 24 24 24 20 15 14 12 11 8 6 5 4 1
MET amplification 11 11 10 9 7 5 4 4 4 4 2 2 2 1 0 0

Supplementary Figure 6. Kaplan-Meier estimate of (a) PFS and (b) OS for participants (n=38) with a detectable *MET* amplification versus no *MET* amplification in baseline plasma. Source data are provided as source data file**.**

Black vertical lines denote censored observations, and the number of participants at risk are indicated below the plots at each time interval. Comparisons were made using a two-sided log-rank test. *MET*, MET proto-oncogene; PFS, progression-free survival; OS, overall survival; HR, hazard ratio; 95% CI, 95% confidence interval.

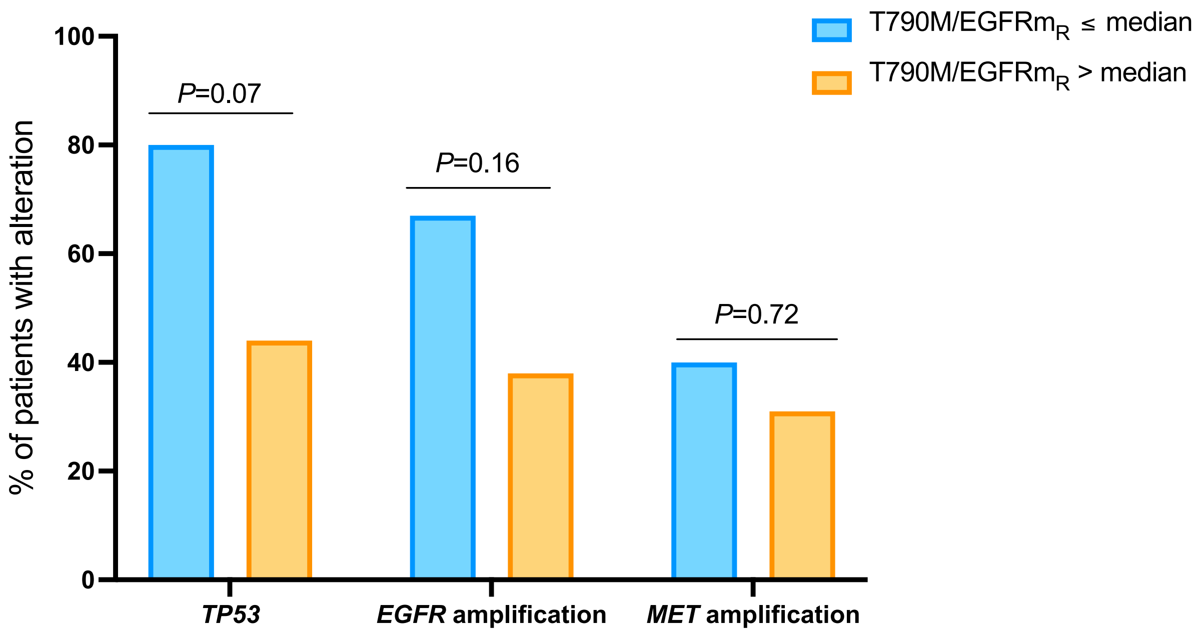

Supplementary Figure 7. Co-alterations detected in baseline plasma in participants with T790M:EGFRm$_R$ ≤median and >median. Source data are provided as source data file. All *P* values are exact, and comparison was made using Fisher's exact test. T790M:EGFRm$_R$, ratio of T790M to epidermal growth factor receptor mutation; *TP53*, tumor protein p53; *EGFR*, epidermal growth factor receptor; *MET*, *MET* proto-oncogene.

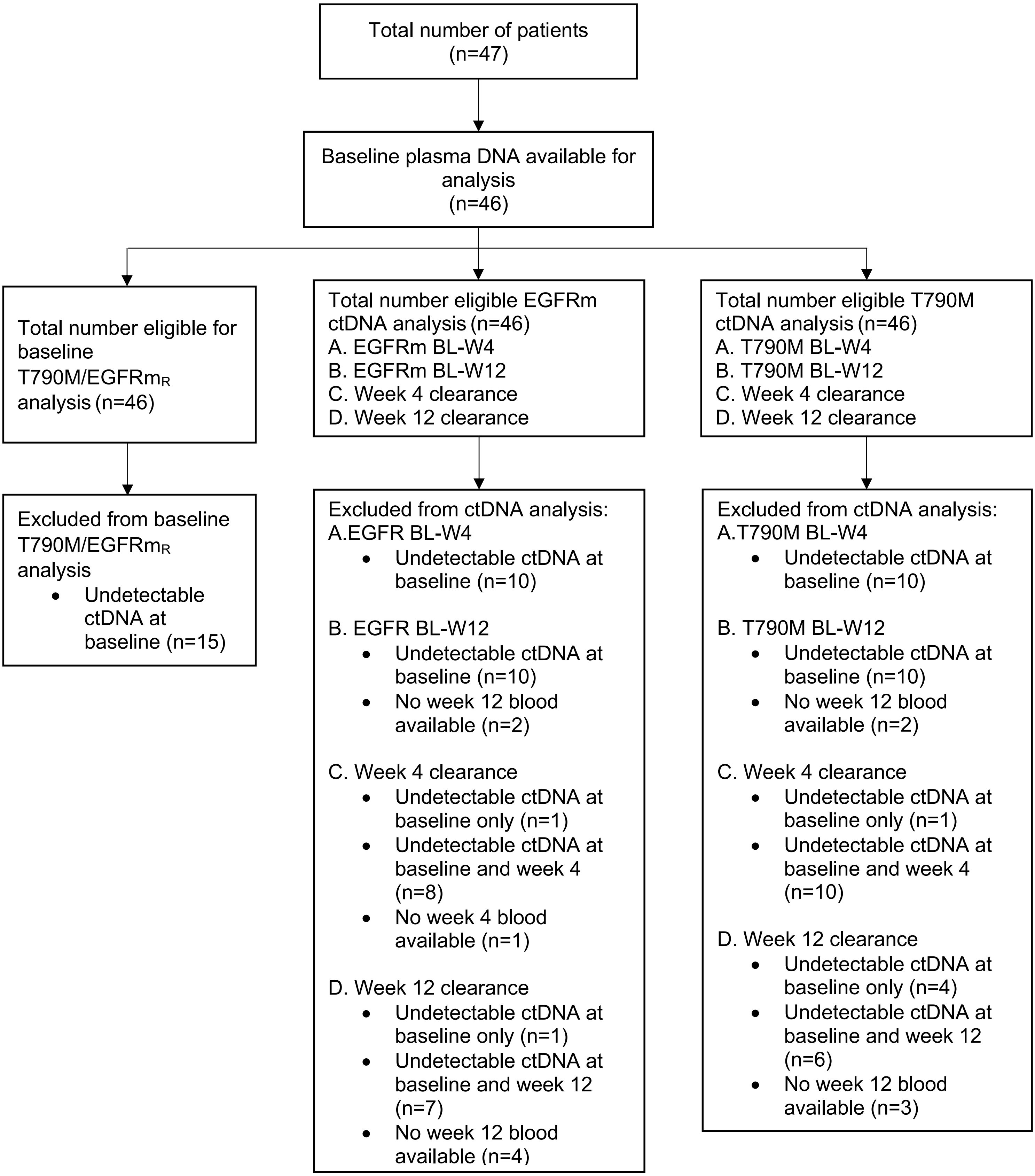
Total number of patients (n=47)
Baseline plasma DNA available for analysis (n=46)
Total number eligible for baseline T790M/EGFRmR analysis (n=46)
Total number eligible EGFRm ctDNA analysis (n=46)
A. EGFRm BL-W4
B. EGFRm BL-W12
C. Week 4 clearance
D. Week 12 clearance
Total number eligible T790M ctDNA analysis (n=46)
A. T790M BL-W4
B. T790M BL-W12
C. Week 4 clearance
D. Week 12 clearance
Excluded from baseline T790M/EGFRmR analysis
• Undetectable ctDNA at baseline (n=15)
Excluded from ctDNA analysis:
A.EGFR BL-W4
• Undetectable ctDNA at baseline (n=10)
B. EGFR BL-W12
• Undetectable ctDNA at baseline (n=10)
• No week 12 blood available (n=2)
C. Week 4 clearance
• Undetectable ctDNA at baseline only (n=1)
• Undetectable ctDNA at baseline and week 4 (n=8)
• No week 4 blood available (n=1)
D. Week 12 clearance
• Undetectable ctDNA at baseline only (n=1)
• Undetectable ctDNA at baseline and week 12 (n=7)
• No week 12 blood available (n=4)
Excluded from ctDNA analysis:
A.T790M BL-W4
• Undetectable ctDNA at baseline (n=10)
B. T790M BL-W12
• Undetectable ctDNA at baseline (n=10)
• No week 12 blood available (n=2)
C. Week 4 clearance
• Undetectable ctDNA at baseline only (n=1)
• Undetectable ctDNA at baseline and week 4 (n=10)
D. Week 12 clearance
• Undetectable ctDNA at baseline only (n=4)
• Undetectable ctDNA at baseline and week 12 (n=6)
• No week 12 blood available (n=3)

Supplementary Figure 8. Consort diagram of plasma samples analyzed from OSCILLATE trial. Source data are provided as source data file**.**

ctDNA, circulating tumor DNA; T790M/EGFRm_R, ratio of T790M to epidermal growth factor receptor mutation; BL-W4, baseline to week 4; BL-W12, baseline to week 12.

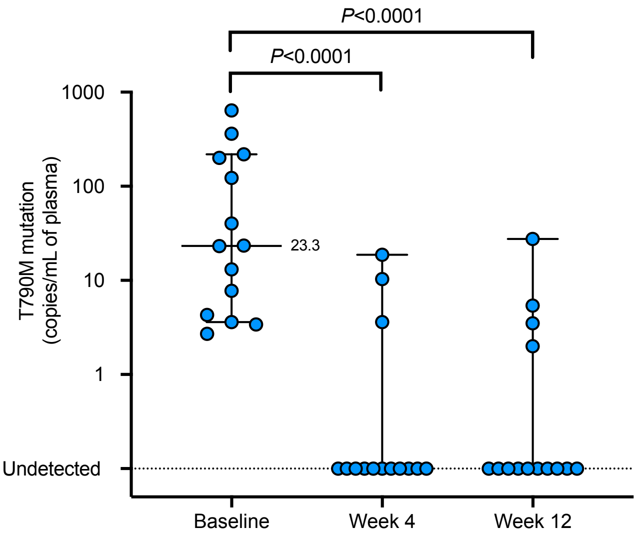

Partial Response

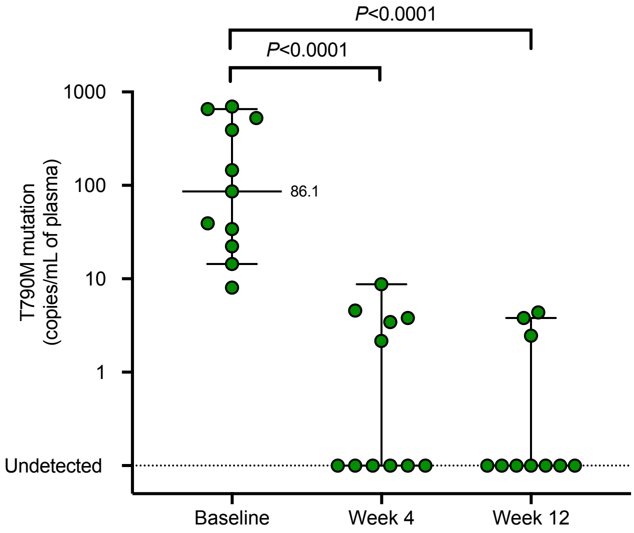

Stable Disease

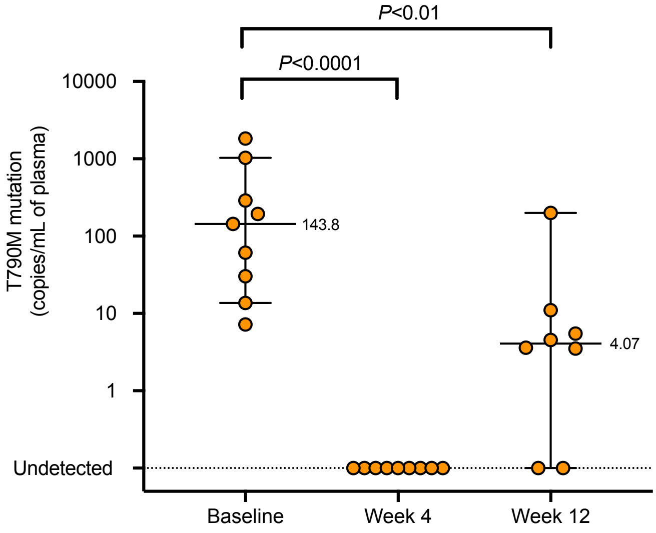

Progressive Disease

Supplementary Figure 9. Dynamics of T790M mutant DNA copies/mL between baseline and week 4 (n=36), and baseline and week 12 (n=34) in participants achieving PR, SD, and PD. Source data are provided as source data file.

Data are presented as median value ± 95% confidence interval. Each dot represents a single participant. Comparison between baseline and week 4, and baseline and week 12 were made using a two-tailed Wilcoxon signed-rank test. All *P* values are exact. PR, partial response; SD, stable disease; PD, progressive disease.

a)

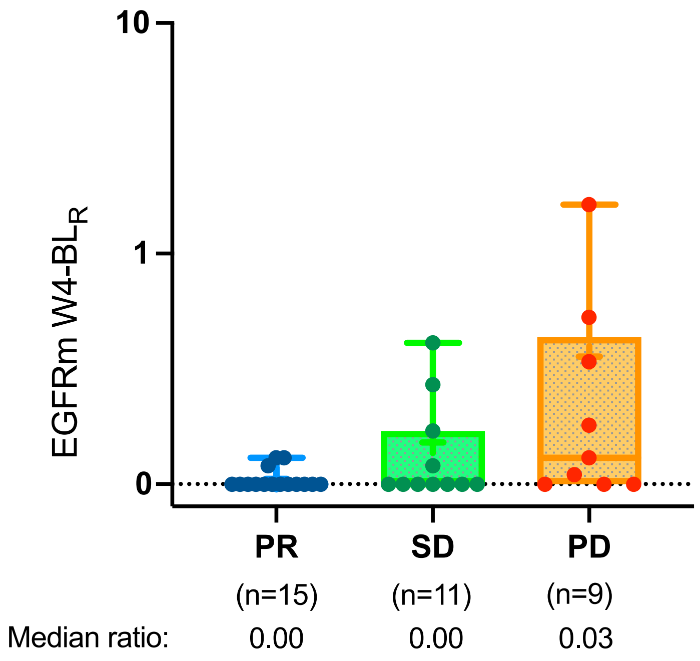

10
1
0
EGFRm W4-BL$_R$
PR
SD
PD
(n=15)
(n=11)
(n=9)
Median ratio:
0.00
0.00
0.03

b)

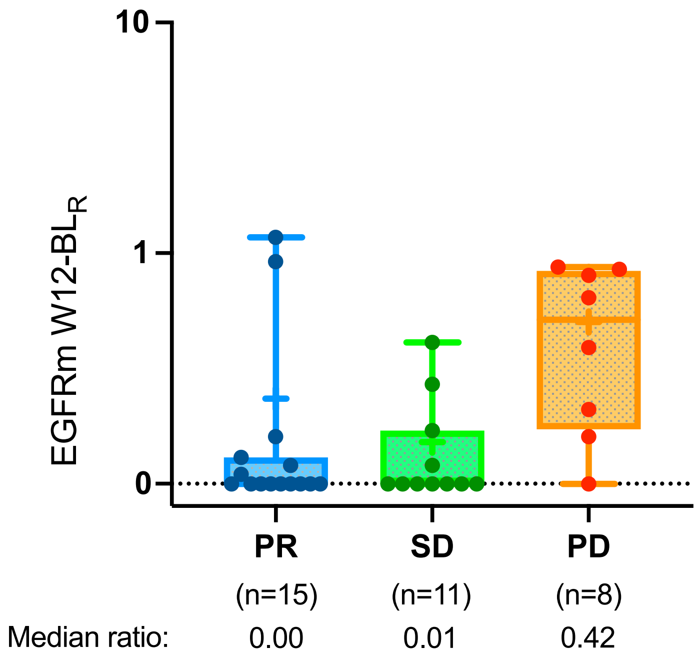

10
1
0
EGFRm W12-BL$_R$
PR
SD
PD
(n=15)
(n=11)
(n=8)
Median ratio:
0.00
0.01
0.42

Supplementary Figure 10. a) EGFRm W4-BLR and b) EGFRm W12-BLR for participants achieving PR, SD, or PD. Source data are provided as source data file.

Horizontal line, boxes, whiskers, and dots indicate mean, quantiles, minima/maxima, and each participant, respectively. W4-$BL_{R,}$ Week 4 to baseline ratio; W12-$BL_{R,}$ Week 12 to baseline ratio; EGFR, epidermal growth factor receptor; PR, partial response; SD, stable disease; PD, progressive disease.

a)

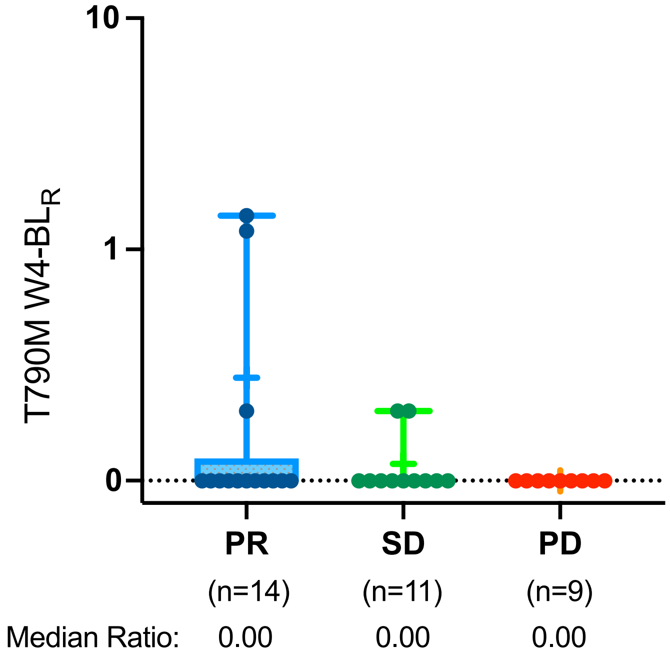
10
1
0
T790M W4-BL_R
PR
SD
PD
(n=14)
(n=11)
(n=9)
Median Ratio:
0.00
0.00
0.00

b)

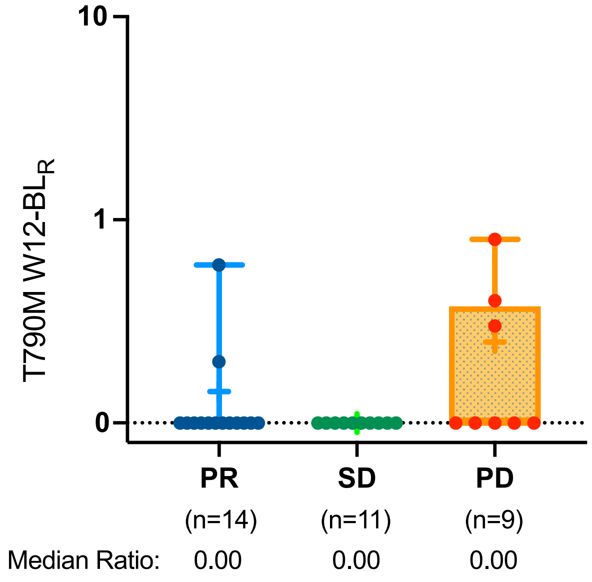
10
1
0
T790M W12-BL_R
PR
SD
PD
(n=14)
(n=11)
(n=9)
Median Ratio:
0.00
0.00
0.00

Supplementary Figure 11. a) T790M W4-BL_R and b) T790M W12-BL_R for participants achieving PR, SD, or PD. Source data are provided as source data file.

Horizontal line, boxes, whiskers, and dots indicate mean, quantiles, minima/maxima, and each participant, respectively. W4-$BL_{R,}$ Week 4 to baseline ratio; PR, partial response; SD, stable disease; PD, progressive disease.

a)

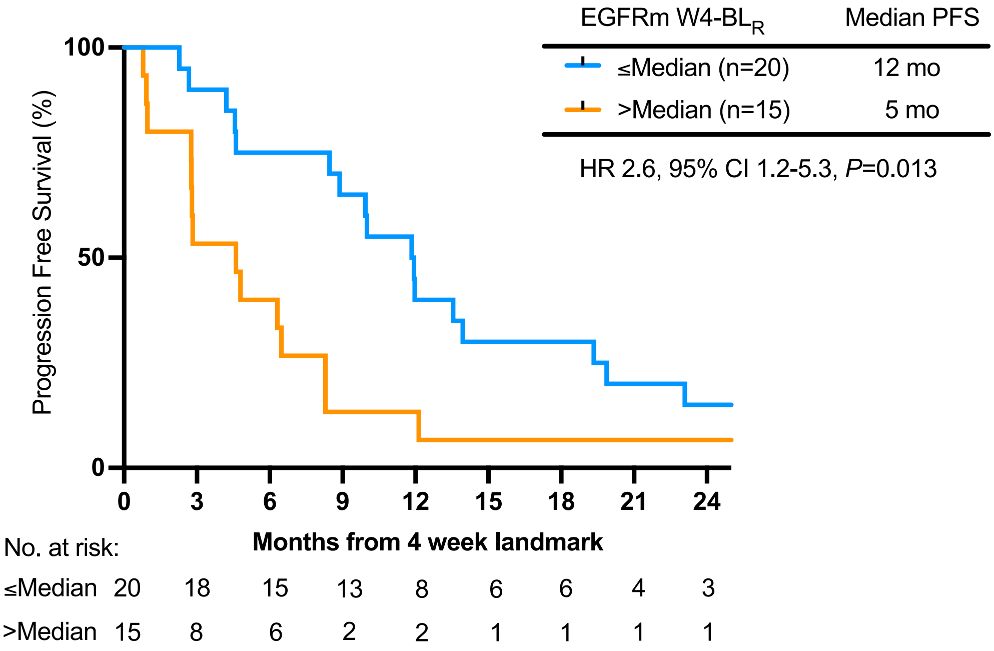

EGFRm W4-BL$_R$
Median PFS
≤Median (n=20)
12 mo
>Median (n=15)
5 mo
HR 2.6, 95% CI 1.2-5.3, *P*=0.013
Progression Free Survival (%)
100
50
0
0 3 6 9 12 15 18 21 24
Months from 4 week landmark
No. at risk:
≤Median 20 18 15 13 8 6 6 4 3
>Median 15 8 6 2 2 1 1 1 1

b)

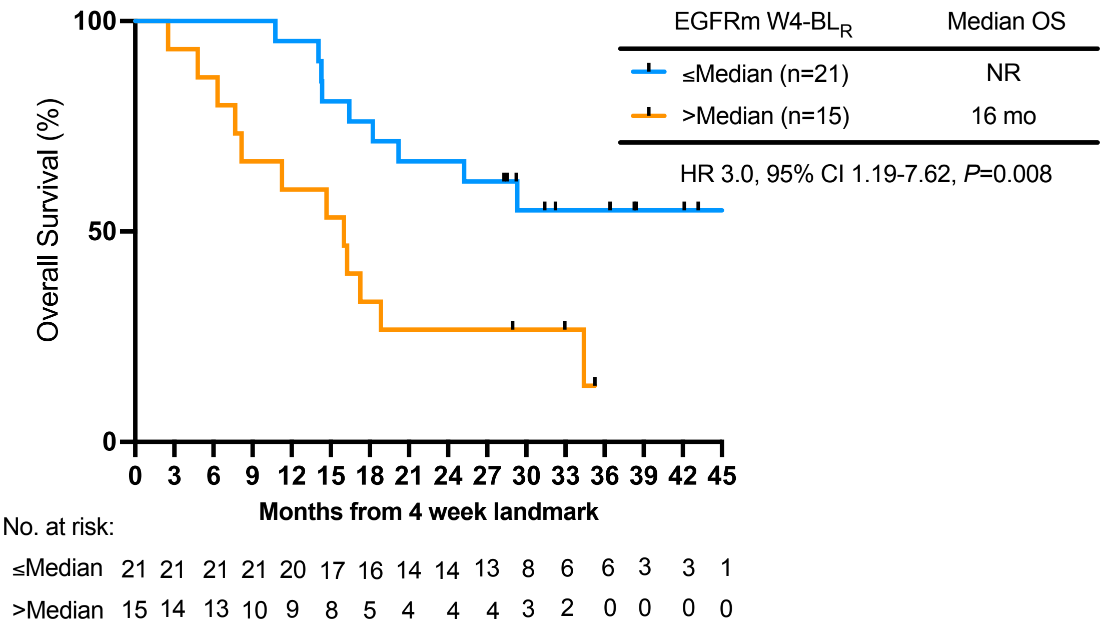

EGFRm W4-BL$_R$
Median OS
≤Median (n=21)
NR
>Median (n=15)
16 mo
HR 3.0, 95% CI 1.19-7.62, *P*=0.008
Overall Survival (%)
100
50
0
0 3 6 9 12 15 18 21 24 27 30 33 36 39 42 45
Months from 4 week landmark
No. at risk:
≤Median 21 21 21 21 20 17 16 14 14 13 8 6 6 3 3 1
>Median 15 14 13 10 9 8 5 4 4 4 3 2 0 0 0 0

Supplementary Figure 12. Kaplan-Meier estimate of a) PFS and b) OS for participants (n=35 and n=36, respectively) stratified according to EGFRm W4-BL_R ≤median and >median. Source data are provided as source data file.

Black vertical lines denote censored observations, and the number of participants at risk are indicated below the plots at each time interval. Comparisons were made using a two-sided log-rank test. EGFRm, epidermal growth factor receptor mutation; W4-BL_R , week 4 to baseline ratio; PFS, progression-free survival; OS, overall survival; HR, hazard ratio; 95% CI, 95% confidence interval.

a)

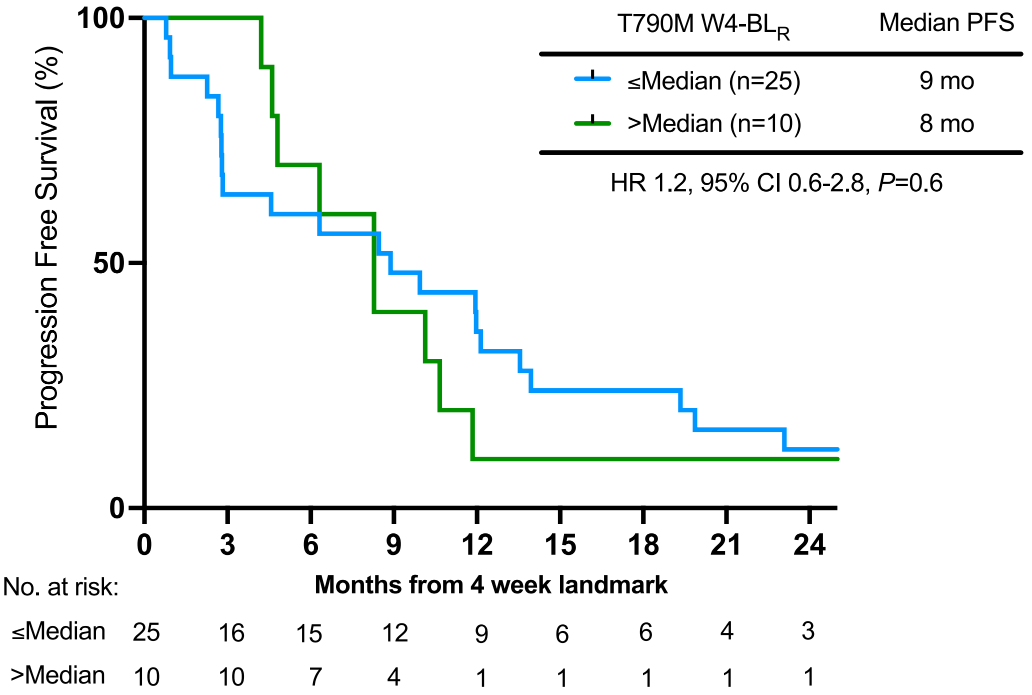

T790M W4-BL$_R$
Median PFS
≤Median (n=25) 9 mo
>Median (n=10) 8 mo
HR 1.2, 95% CI 0.6-2.8, *P*=0.6
Progression Free Survival (%)
100
50
0
0 3 6 9 12 15 18 21 24
Months from 4 week landmark
No. at risk:
≤Median 25 16 15 12 9 6 6 4 3
>Median 10 10 7 4 1 1 1 1 1

b)

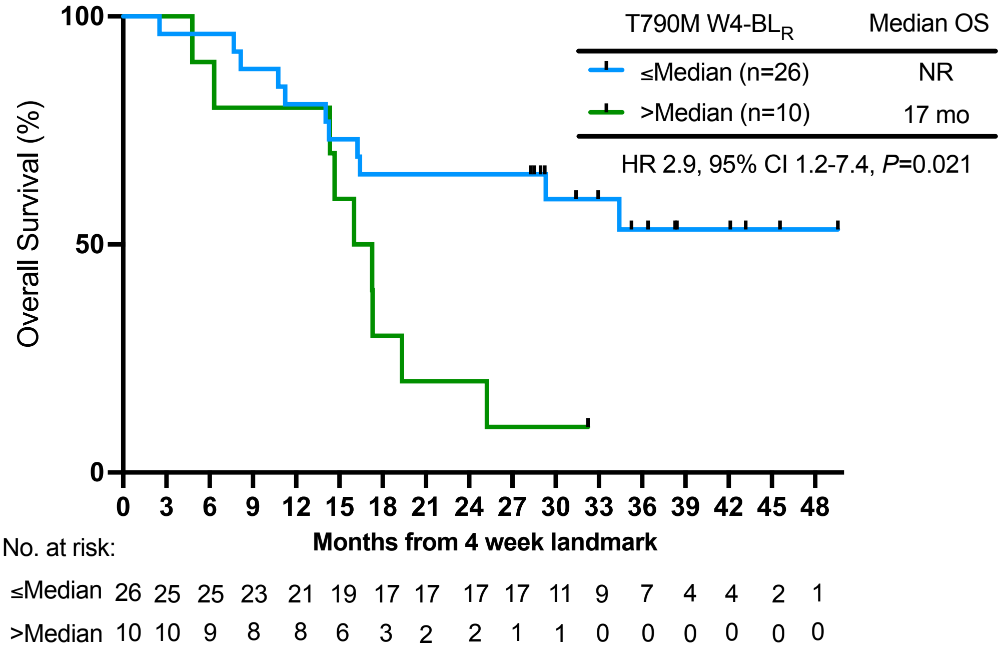

T790M W4-BL$_R$
Median OS
≤Median (n=26) NR
>Median (n=10) 17 mo
HR 2.9, 95% CI 1.2-7.4, *P*=0.021
Overall Survival (%)
100
50
0
0 3 6 9 12 15 18 21 24 27 30 33 36 39 42 45 48
Months from 4 week landmark
No. at risk:
≤Median 26 25 25 23 21 19 17 17 17 17 11 9 7 4 4 2 1
>Median 10 10 9 8 8 6 3 2 2 1 1 0 0 0 0 0 0

Supplementary Figure 13. Kaplan-Meier estimate of a) PFS and b) OS for participants (n=35 and n=36, respectively) stratified according to T790M W4-BL_R ≤median and >median. Source data are provided as source data file.

Black vertical lines denote censored observations, and the number of participants at risk are indicated below the plots at each time interval. Comparisons were made using a two-sided log-rank test. W4-BL_R , week 4 to baseline ratio; PFS, progression-free survival; OS, overall survival; HR, hazard ratio; 95% CI, 95% confidence interval.

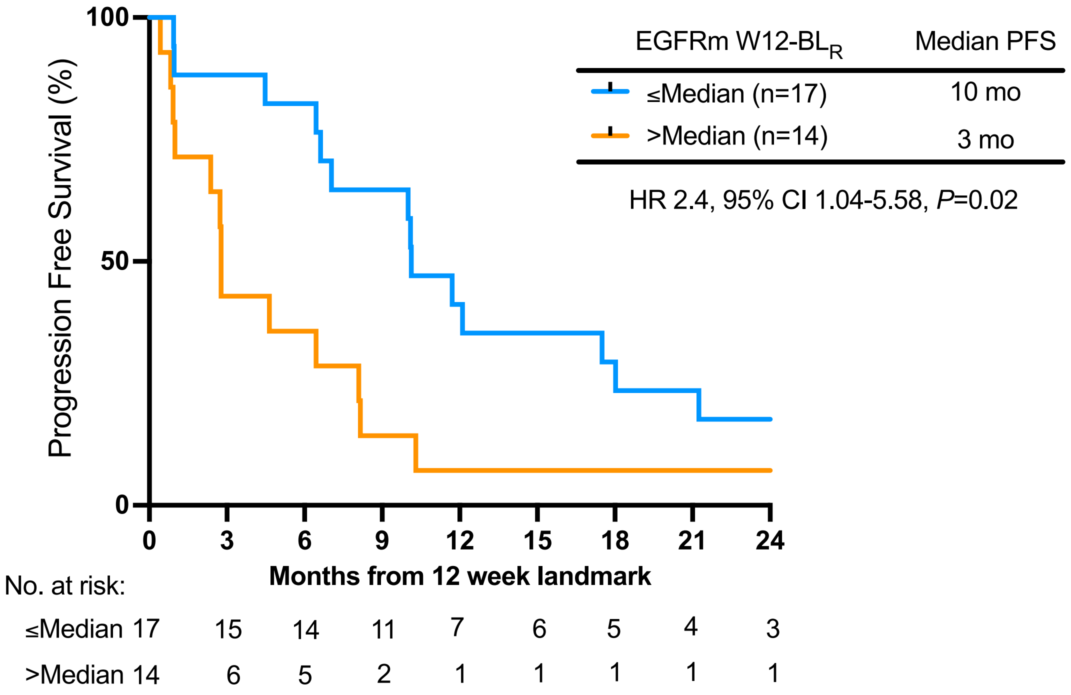

Supplementary Figure 14. Kaplan-Meier estimate of PFS for participants (n=31) stratified according to EGFRm W12-BL_R ≤median and >median. Source data are provided as source data file.

The number of participants at risk are indicated below the plots at each time interval. Comparisons were made using a two-sided log-rank test. EGFRm, epidermal growth factor receptor mutation; W12-BL_R , week 12 to baseline ratio; PFS, progression-free survival; HR, hazard ratio; 95% CI, 95% confidence interval.

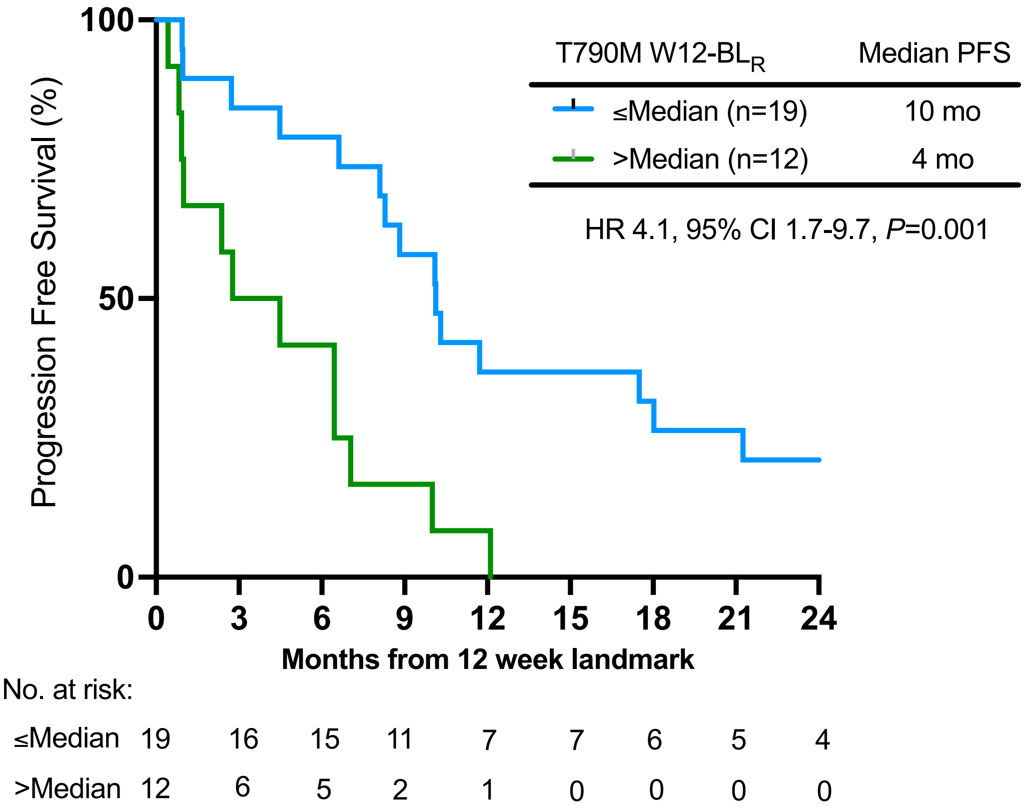

Supplementary Figure 15. Kaplan-Meier estimate of PFS for participants (n=31) stratified according to T790M W12-BL$_R$ ≤median and >median. Source data are provided as source data file**.**

The number of participants at risk are indicated below the plots at each time interval. Comparisons were made using a two-sided log-rank test. EGFRm, epidermal growth factor receptor mutation; W12-BL$_R$, week 12 to baseline ratio; PFS, progression-free survival, HR, hazard ratio; 95% CI, 95% confidence interval.

a)

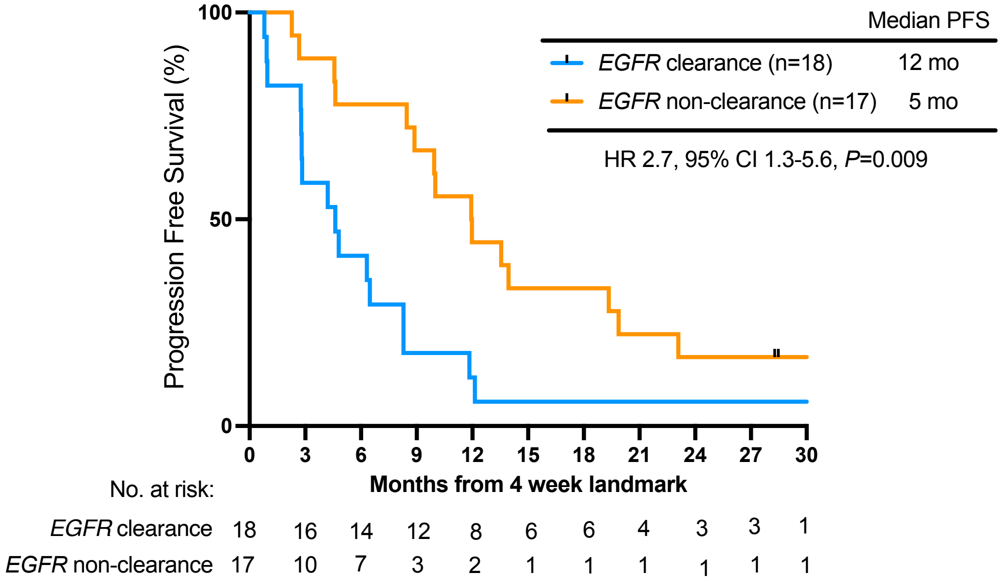
Median PFS
EGFR clearance (n=18) 12 mo
EGFR non-clearance (n=17) 5 mo
HR 2.7, 95% CI 1.3-5.6, P=0.009
Progression Free Survival (%)
100
50
0
0 3 6 9 12 15 18 21 24 27 30
Months from 4 week landmark
No. at risk:
EGFR clearance 18 16 14 12 8 6 6 4 3 3 1
EGFR non-clearance 17 10 7 3 2 1 1 1 1 1 1

b)

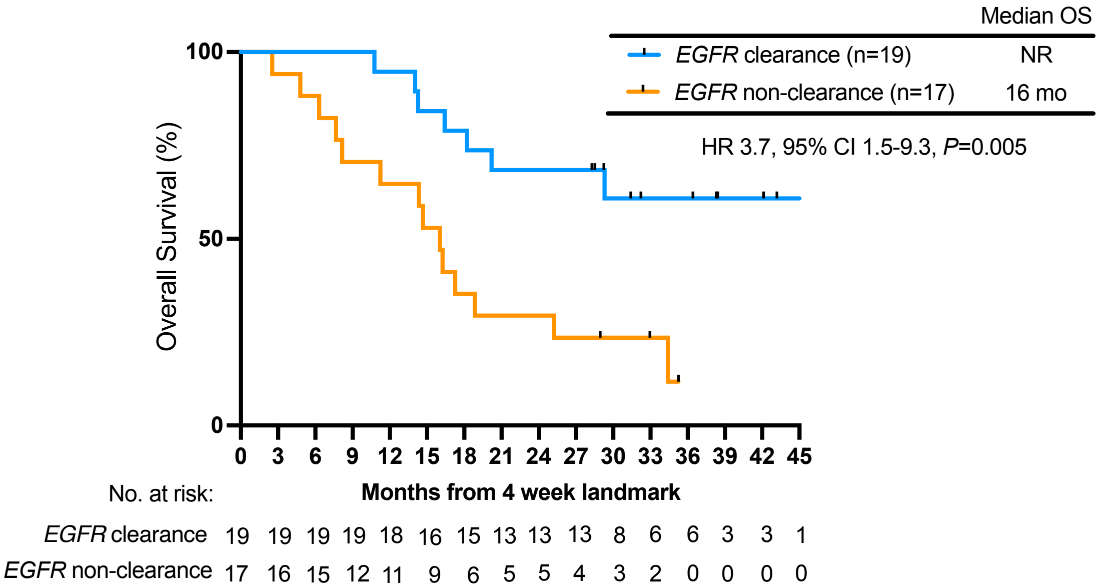
Median OS
EGFR clearance (n=19) NR
EGFR non-clearance (n=17) 16 mo
HR 3.7, 95% CI 1.5-9.3, P=0.005
Overall Survival (%)
100
50
0
0 3 6 9 12 15 18 21 24 27 30 33 36 39 42 45
Months from 4 week landmark
No. at risk:
EGFR clearance 19 19 19 19 18 16 15 13 13 13 8 6 6 3 3 1
EGFR non-clearance 17 16 15 12 11 9 6 5 5 4 3 2 0 0 0 0

Supplementary Figure 16. Kaplan-Meier estimate of a) PFS and b) OS for participants (n=35 and n=36, respectively) stratified according to clearance versus non-clearance of EGFRm DNA (copies/mL) by week 4. Source data are provided as source data file**.**

Black vertical lines denote censored observations, and the number of participants at risk are indicated below the plots at each time interval. Comparisons were made using a two-sided log-rank test. EGFRm, epidermal growth factor receptor mutation; PFS, progression-free survival; OS, overall survival; HR, hazard ratio; 95% CI, 95% confidence interval.

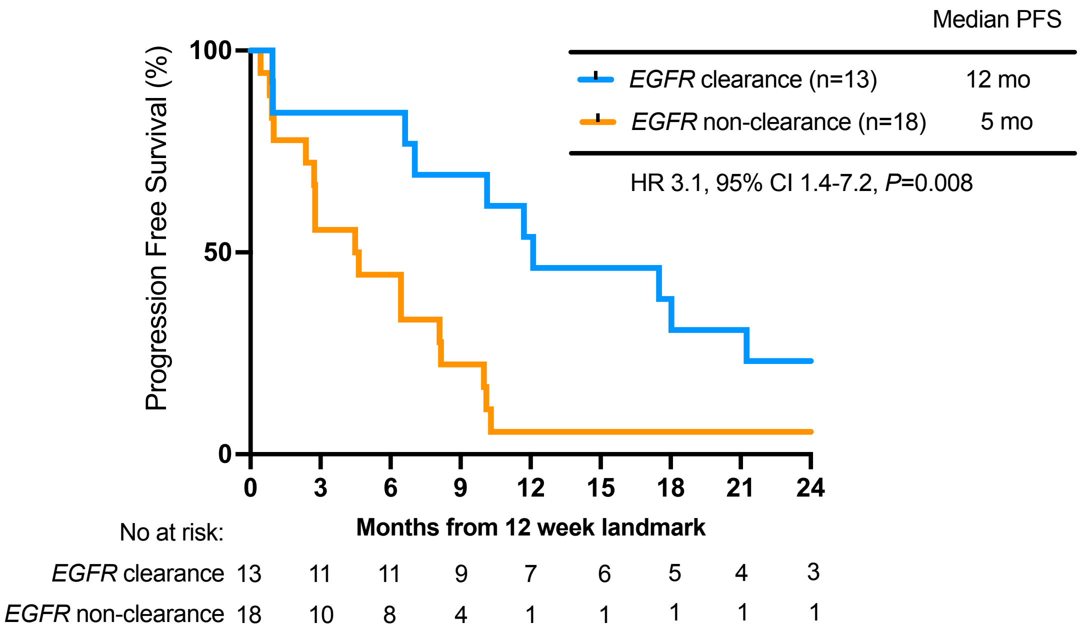

Supplementary Figure 17. Kaplan-Meier estimate of PFS for participants (n=31) stratified according to clearance versus non-clearance of EGFRm DNA (copies/mL) by week 12. Source data are provided as source data file**.**

The number of participants at risk are indicated below the plots at each time interval. Comparisons were made using a two-sided log-rank test. EGFRm, epidermal growth factor receptor mutation; PFS, progression-free survival; HR, hazard ratio; 95% CI, 95% confidence interval.

a)

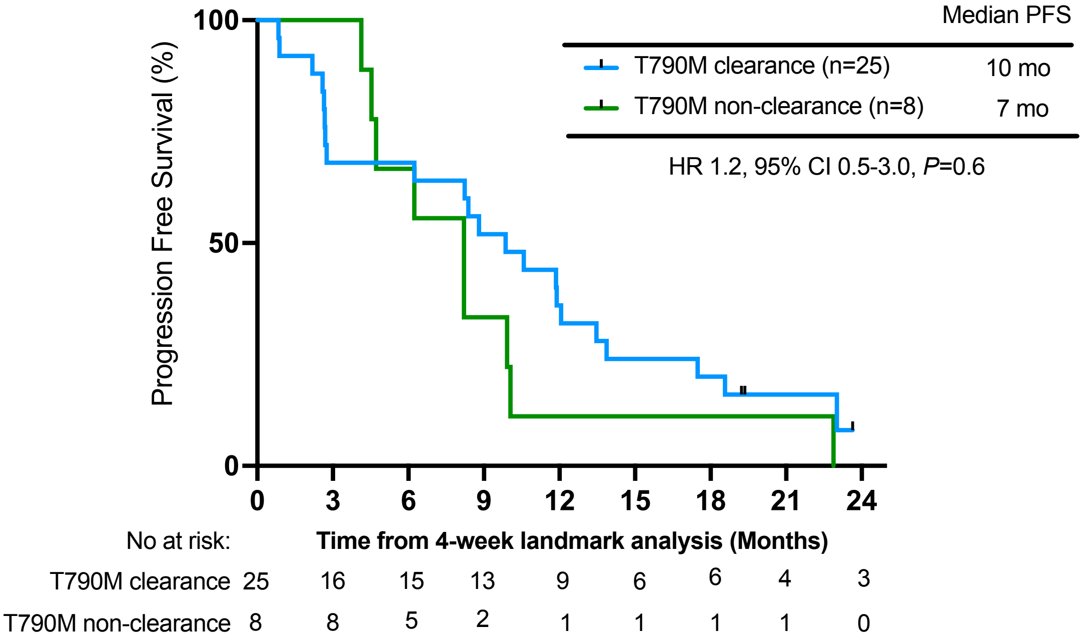
Median PFS
T790M clearance (n=25)
10 mo
T790M non-clearance (n=8)
7 mo
HR 1.2, 95% CI 0.5-3.0, *P*=0.6
Progression Free Survival (%)
100
50
0
0 3 6 9 12 15 18 21 24
Time from 4-week landmark analysis (Months)
No at risk:
T790M clearance 25 16 15 13 9 6 6 4 3
T790M non-clearance 8 8 5 2 1 1 1 1 0

b)

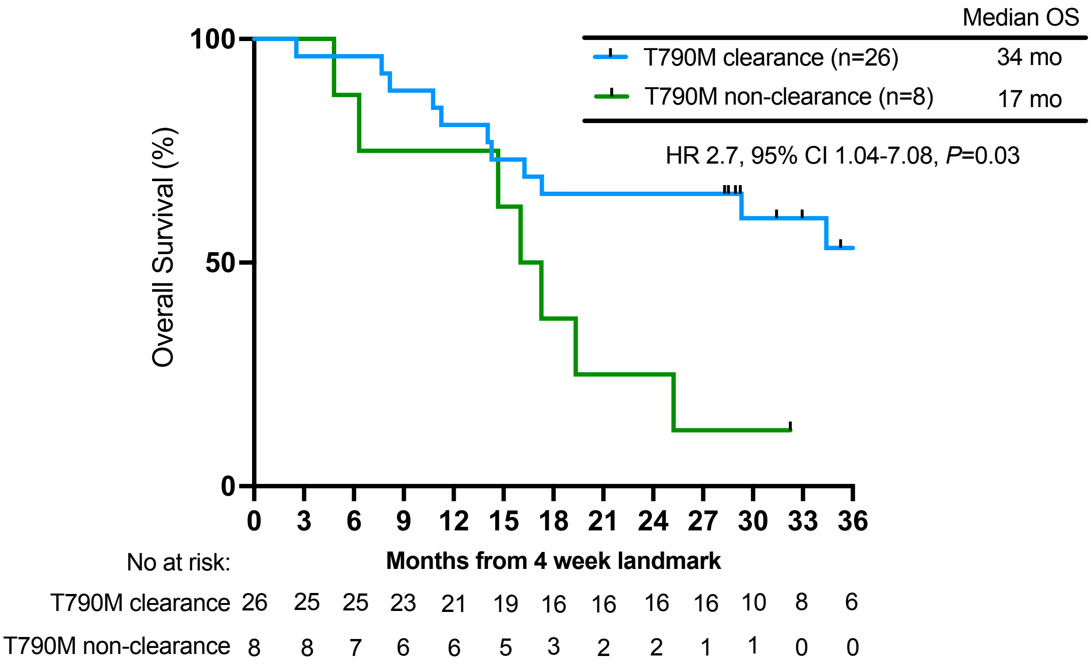
Median OS
T790M clearance (n=26)
34 mo
T790M non-clearance (n=8)
17 mo
HR 2.7, 95% CI 1.04-7.08, *P*=0.03
Overall Survival (%)
100
50
0
0 3 6 9 12 15 18 21 24 27 30 33 36
Months from 4 week landmark
No at risk:
T790M clearance 26 25 25 23 21 19 16 16 16 16 10 8 6
T790M non-clearance 8 8 7 6 6 5 3 2 2 1 1 0 0

Supplementary Figure 18. Kaplan-Meier estimate of a) PFS and b) OS for participants (n=33 and n=34, respectively) stratified according to clearance versus non-clearance of T790M DNA (copies/mL) by week 4. Source data are provided as source data file**.** Black vertical lines denote censored observations, and the number of participants at risk are indicated below the plots at each time interval. Comparisons were made using a two-sided log-rank test. PFS, progression-free survival; OS, overall survival; HR, hazard ratio; 95% CI, 95% confidence interval.

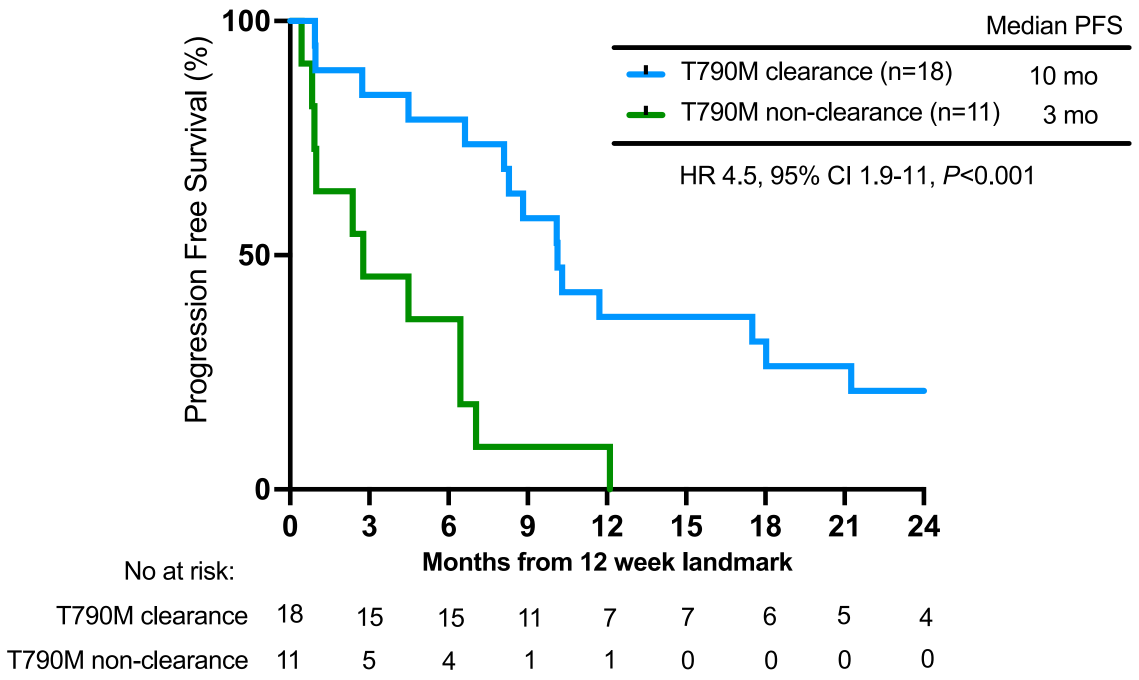

Supplementary Figure 19. Kaplan-Meier estimate of PFS for participants (n=30) stratified according to clearance versus non-clearance of T790M mutant DNA (copies/mL) by week 12. Source data are provided as source data file**.**

The number of participants at risk are indicated below the plots at each time interval. Comparisons were made using a two-sided log-rank test. PFS, progression-free survival; HR, hazard ratio; 95% CI, 95% confidence interval

Supplementary Table 1. Concordance of baseline plasma *EGFR*m and T790M mutation detection between ddPCR and targeted sequencing assays. Source data are provided as source data file**.**

Mutation	Detected via ddPCR only	Detected via targeted sequencing only	Detected via ddPCR and targeted sequencing	Total (n)
Activating *EGFR* (includes ex19del and L858R)	2	1	33	36
Ex19del	2	1	20	23
T790M	6	1	28	35

Abbreviations: ddPCR, digital droplet PCR; EGFRm, epidermal growth factor receptor mutation; Ex19del, exon 19 deletion.

Study Title

Phase 2 trial of alternating osimertinib with gefitinib in patients with EGFR-T790M mutation positive advanced non-small cell lung cancer – Oscillate.

NHMRC CTC protocol number

CTC 0152 / ALTG 16/005

Protocol version number and date

Version 1.2, dated 4 May 2017

**Australian Sponsor:**	The University of Sydney<br>NSW 2006 Australia<br><br>This study is a collaboration between the Australasian Lung Cancer Trials Group (ALTG) and the NHMRC Clinical Trials Centre (NHMRC CTC), University of Sydney.
**Study Chair:**	Benjamin Solomon<br>Peter MacCallum Cancer Centre<br>Melbourne, VIC
**Coordinating Centre:**	NHMRC Clinical Trials Centre<br>92-94 Parramatta Road<br>Camperdown NSW 2050<br>Telephone: 61-2-9562-5000<br>Fax: 61-2-9565-1863<br>Email: oscillate@ctc.usyd.edu.au
**Study Statistician:**	Chris Brown, CTC
**CTC Clinical Lead:**	Martin Stockler, CTC
**Project Manager:**	Hannora Jurkovic, CTC

CONFIDENTIAL

Protocol Development Working Party

The following individuals also contributed to the design and development of this protocol:

Name	Position	Organisation
Ann Livingstone	ALTG Associate Oncology Program Manager	CTC
Dr Peey-Sei Kok<br>Dr Lavinia Tan	ALTG Clinical Research Fellow<br>Lung Cancer Fellow	CTC<br>Peter MacCallum Cancer Centre
Dr Sonia Yip	Senior Translational Research Fellow and Manager	CTC

Abbreviations

AE	Adverse event
ALT	Alanine aminotransferase
ALTG	Australasian Lung Cancer Trials Group
AST	Aspartate aminotransferase
ATP	Adenosine triphosphate
AUC	Area under the concentration-time curve
cfDNA	Cell free DNA
CI	Confidence interval
CrCl	Creatinine Clearance
CR	Complete response
CRF	Case report form
CT	Computed tomography
del19	Deletion 19
dPCR	Digital Polymerase chain reaction
ECG	Electrocardiogram
ECOG	Eastern Cooperative Oncology Group
EGFR	Epidermal Growth Factor receptor
eCRF	Electronic Case report form
ESMO	European Society of Medical Oncology
FFPE	Formalin fixed paraffin embedded
GCP	Good Clinical Practice
HIV	Human immunodeficiency virus
HREC	Human research ethics committee
ICH	International Conference on Harmonization
ILD	Interstitial lung disease
IV	Intravenous(ly)
NCCN	National Comprehensive Cancer Network
MAPK	Mitogen-activated protein kinase
MET	Mesenchymal-epithelial transition
MRI	Magnetic resonance imaging
NCI CTCAE	National Cancer Institute Common Terminology Criteria for Adverse Events
NGS	Next generation sequencing
NHMRC CTC	National Health and Medical Research Council, Clinical Trials Centre
NSCLC	Non-small cell lung cancer
OR	Objective response
OTRR	Objective tumour response rate
OS	Overall survival
PCR	Polymerase chain reaction
PD	Progressive disease
PFS	Progression-free survival
PI3K	Phosphatidylinositol-3-kinase
PK	Pharmacokinetic
PR	Partial response
QTc	The time between the start of the Q wave and the end of the T wave corrected for heart rate
RECIST	Response Evaluation Criteria in Solid Tumours
SAE	Serious adverse event
SD	Stable disease
SUSAR	Suspected unexpected serious adverse reaction
TKIs	Tyrosine kinase inhibitors
TMC	Trial Management Committee
ULN	Upper limit of normal

Table of Contents

SYNOPSIS AND SCHEMA 6
1 BACKGROUND 8
1.1 Epidermal growth factor receptor (EGFR) mutation positive non-small cell lung cancers .. 8
1.2 Treatment of EGFR mutation positive advanced non-small cell lung cancer 8
1.3 Acquired resistance to EGFR Tyrosine Kinase Inhibitors 9
1.4 Treatment of EGFR-T790M positive non-small cell lung cancer 9
1.5 Osimertinib (AZD9291) 10
1.6 EGFR T790M mutation - plasma cfDNA testing 10
1.7 Rationale for this study 11
2 AIM AND OBJECTIVES 12
3 DESIGN 12
4 STUDY POPULATION 12
4.1 Target Population 12
4.2 Inclusion Criteria 12
4.3 Exclusion Criteria 13
4.4 Study Enrolment 14
4.4.1 Screening 14
4.4.2 Registration 14
5 TREATMENT PLAN 14
5.1 Treatments 14
5.1.1 Required Background Treatment 15
5.2 Administration of treatment 15
5.3 Dose Modifications 15
5.3.1 Dose Modifications for Adverse Events 16
5.4 Post-study treatment 20
5.5 Concomitant Medications 20
5.5.1 Anticancer or experimental, radiotherapy or surgical therapy 20
5.5.2 Hematopoietic Growth Factors and Blood Products 21
5.5.3 Contra-indicated medications 21
5.5.4 Use with Caution 21
5.5.5 Guidance regarding potential interactions with specific concomitant medications/devices 21
5.5.6 Concomitant Medication Reporting 22
5.5.7 Restrictions for sexually active participants 22
5.6 Compliance 22
5.7 Study Treatment Discontinuation 22
5.8 Treatment beyond progression 23
6 ASSESSMENT PLAN 24
6.1 Schedule of Assessments 24
6.2 Details of Assessments 26
6.2.1 Screening phase 26
6.2.2 Treatment Phase 26
6.2.3 Clinical assessment 26
6.2.1 Imaging 26
6.2.4 Blood Collection 27
6.2.5 EGFR-T790M mutation testing/ liquid biopsy 27
6.2.6 Tissue samples 27
7 OUTCOMES, ENDPOINTS AND OTHER MEASUREMENTS 27
7.1 Progression free survival (PFS) at 12 months 27
7.2 Feasibility of alternating osimertinib and gefitinib 28
7.3 Time to progression (TTP) 28

7.4 Objective tumour response rate (OTRR = CR and/or PR) 28
7.5 Overall survival (OS) 28
7.6 Adverse events 28
7.7 Tertiary/correlative measures 28
8 SAFETY REPORTING 29
8.1 Definitions 29
8.2 Recording Adverse Events 30
8.3 Pregnancy 30
8.4 Reporting of Serious Adverse Events (including SUSARs) 31
9 CENTRAL REVIEW 31
9.1 Imaging 31
10 CENTRAL STORAGE OF BIOSPECIMENS 31
10.1 Central Tissue Collection 31
10.2 Central Blood Collection 31
11 DRUG SUPPLY AND ACCOUNTABILITY 31
11.1 Osimertinib 31
11.1.1 Formulation/ packaging/ storage 31
11.2 Gefitinib 32
11.2.1 Formulation/ packaging/ storage 32
11.3 Osimertinib and gefitinib supply 32
11.4 Drug Accountability 32
12 STATISTICAL CONSIDERATIONS 32
12.1 Sample Size 32
12.2 Statistical Analysis 32
12.3 Interim analyses 33
13 STUDY ORGANISATION and COMMITTEES 33
13.1 Study coordination 33
13.2 Trial Management Committee 33
14 ADMINISTRATIVE ASPECTS 33
14.1 Ethics and regulatory compliance 33
14.2 Confidentiality 33
14.3 Protocol amendments 34
14.4 Data Handling and Record Keeping 34
14.5 Study Monitoring 34
14.6 Audit and Inspection 34
14.7 Clinical Study Report 35
14.8 Publication Policy 35
15 PROTOCOL AMENDMENTS 35
16 REFERENCES 36
17 APPENDICES 39
17.1 Appendix 1 - Cockroft-Gault formula 39
17.2 Appendix 2 – Guidance regarding potential interactions with concomitant medications .. 40
17.3 Appendix 3 - RECIST v 1.1 44
17.4 Appendix 4 - Common Terminology Criteria for Adverse Events (CTCAE) 47
17.5 Appendix 5 – ECOG Performance status scales 48

SYNOPSIS AND SCHEMA

**Background**	Activating mutations of the epidermal growth factor receptor (EGFR) are key drivers of non-small cell lung cancer (NSCLC) in approximately 10-15% of Western patients and 30-35% of Asian patients. Patients with common activating mutations (L858R and del19) typically have objective tumour response rates (OTRR) of 56-74% and median progression free survival (PFS) of 9-13 months with first-generation EGFR tyrosine kinase inhibitors (TKI) such as erlotinib or gefitinib. The most common mechanism of acquired resistance to first generation EGFR TKI is the T790M mutation (50-60% of cases). Osimertinib is an irreversible EGFR TKI effective against the T790M resistance mutation with OTRR of 51-71% in T790M positive disease. However, acquired resistance invariably develops, and median PFS is approximately 10 months. Mechanisms of resistance include C797S mutations and loss of T790M. Novel strategies that prevent or delay resistance to osimertinib are likely to enhance its durability of response and clinical benefit in EGFR-T790M positive NSCLC.
**Aims**	To determine the efficacy, safety, and feasibility of alternating osimertinib and gefitinib in patients with EGFR-T790M mutation positive NSCLC.
**Objectives**	To determine the
**Primary**	1. PFS rate at 12 months according to RECIST v1.1
**Secondary**	2. feasibility of alternating osimertinib and gefitinib<br>3. time to progression (TTP) and PFS time<br>4. objective tumour response rate (OTRR=CR+PR, RECIST 1.1)<br>5. overall survival (OS)<br>6. frequency and severity of adverse events (CTCAE v4.03)
**Tertiary correlative**	7. changes in plasma cfDNA levels for activating EGFR mutations and T790M over time<br>8. relationships between changes in plasma cfDNA levels for activating EGFR mutations, T790M, and OTRR<br>9. mechanisms of resistance in patients progressing on alternating osimertinib and gefitinib as identified in cfDNA (or where possible tumour biopsy)
**Design**	Open-label, single arm, multi-centre, phase 2 trial with safety run in.
**Population**	Adults with advanced, EGFR mutated NSCLC, acquired resistance to first or second generation EGFR TKIs, and mutation of T790M.
**Study treatments**	Osimertinib 80mg daily for 8 weeks (2 cycles), then gefitinib 250mg daily for 4 weeks alternating with osimertinib 80mg daily for 4 weeks (i.e. alternating 4 weekly cycles of each drug) until disease progression or prohibitive toxicity.
**Assessments**	Clinical assessments before each cycle of osimertinib or gefitinib. CT chest, abdomen and pelvis (and brain if required) at baseline, week 8, then every 8 weeks until disease progression. Plasma collections at baseline and at the start of each 4-week cycle.
**Statistical considerations**	A total of 45 participants provides 90% power, with a 1-sided type 1 error rate of 10%, to distinguish the observed proportion alive and progression free at 12 months from true rates of 45% (not worthy of pursuit) and 65% (worthy of pursuit) using a Simon, 2-stage, minimax design allowing for 4 ineligible or inevaluable participants.

Study Schema

Eligibility
EGFR –T790M positive advanced lung cancer progressed after first or second generation TKI.

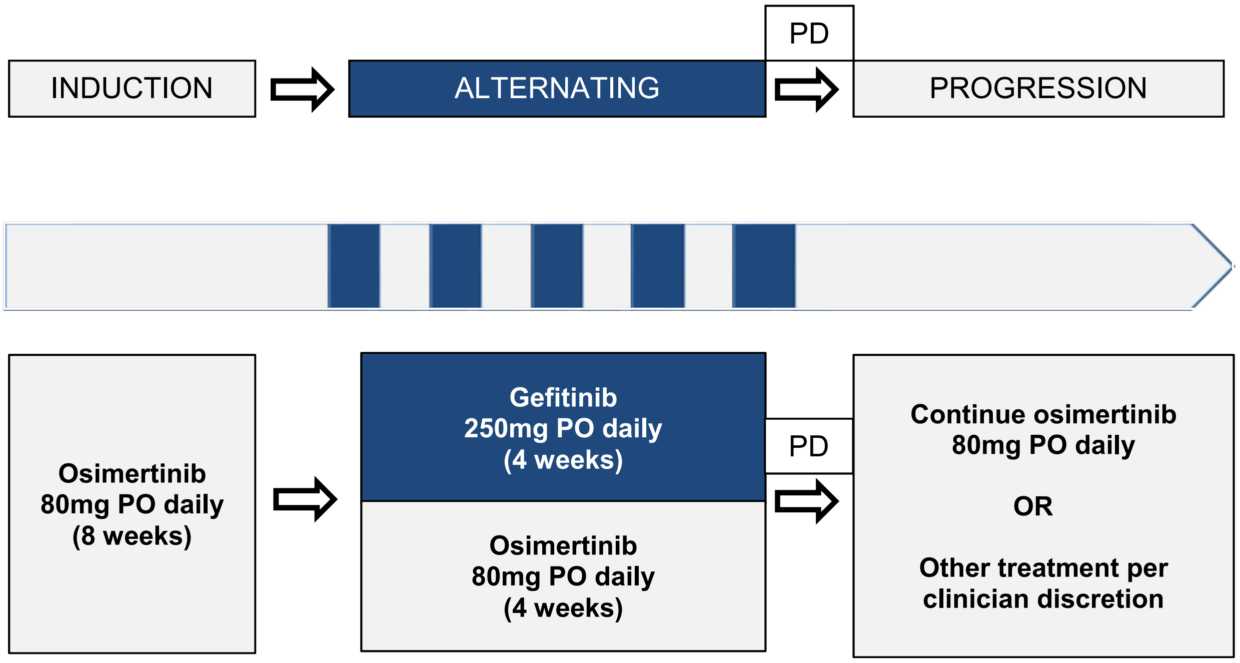

Primary Endpoint:
PFS at 12 months

Secondary Endpoints:
- TTP, PFS
- OTRR
- OS
- Safety

Tertiary Endpoints
- Changes in plasma EGFR mutation and T790M cfDNA over time and with response per imaging
- Mechanism of resistance on patients progressing on alternating treatment.

N= 45
Estimated study duration of 3 years = 2 years accrual plus 1 year additional minimum follow up.

1 BACKGROUND

1.1 *Epidermal growth factor receptor (EGFR) mutation positive non-small cell lung cancers*

Lung cancer remains the leading cause of cancer-related mortality with approximately 13% of all new cancers in 2008 with nearly 1 in 5 (1.6 million, 19% of total) cancer deaths.(1, 2) Approximately 85% of all lung cancer cases are non-small cell lung cancer (NSCLC), the majority present with locally advanced or metastatic disease. (3) Adenocarcinoma, the most common type of lung cancer has a higher than 50% estimated frequency of actionable oncogenic driver mutations.(4)

Activating EGFR mutations are key drivers in NSCLC in approximately 10-15% of Western patients and 30-35% of Asian patients.(5) The clinically relevant and most frequent EGFR mutations are in-frame deletions/insertions of exon 19 (40-50%) and L858R mutation of exon 21 (30-40%). Lung cancers with EGFR mutations depend on EGFR signalling for growth and survival and confer sensitivity to treatment with EGFR tyrosine kinase inhibitors.(6) Hence, it is now part of routine clinical practice to screen for EGFR mutation in newly diagnosed patients with NSCLC especially those with adenocarcinoma histology.

1.2 *Treatment of EGFR mutation positive advanced non-small cell lung cancer*

Multiple randomized clinical trials has shown that gefitinib or erlotinib have superior efficacy as compared with standard chemotherapy when given in the first line setting in patients with EGFR mutation positive advanced NSCLC. (7)

The Iressa Pan-Asia Study (IPASS) enrolled Asian patients with lung adenocarcinoma who were never smokers or light smokers, independent of their EGFR mutation status to randomly receive standard carboplatin plus paclitaxel versus gefitinib as a first-line treatment. The study met its primary objective of demonstrating non-inferiority and showed superiority of gefitinib as compared with carboplatin-paclitaxel for progression free survival (PFS) (hazard ratio [HR] of 0.74; 95% confidence interval [CI] 0.65-0.85; p<0.001). In a subgroup analysis of 261 patients with EGFR mutations, median PFS was significantly longer at 9.5 months for those receiving gefitinib compared to 6.3 months for those receiving carboplatin-paclitaxel (HR for progression 0.48; 95% CI 0.36-0.64; p<0.0001). In contrast, 176 patients in the mutation-negative subgroup had significantly shorter PFS among patients receiving gefitinib than those receiving carboplatin-paclitaxel (HR 2.85; 95% CI 2.05-3.98; p<0.0001).(8)

The WJTOG3405 study enrolled Japanese patients with EGFR mutations on an open label, phase 3 study randomising patients to gefitinib or docetaxel plus cisplatin. Patients in the gefitinib group had a longer median PFS of 9.2 months versus 6.3 months in the chemotherapy group (HR 0.49; 95% CI 0.34-0.71; p<0.0001).(9)

The NEJ002 study compared gefitinib with carboplatin and paclitaxel in EGFR mutation positive patients. Patients in the gefitinib group had a longer median PFS (10.8 months vs 5.4 months; HR 0.30, 95% CI 0.22-0.41; p<0.001) and a higher response rate (73.7% vs 30.7%; p<0.001) compared to patients who received chemotherapy.(10)

The EURTAC multicentre, open label, phase 3 trial randomized 173 patients with EGFR mutation positive advanced NSCLC to receive erlotinib or standard chemotherapy with cisplatin or carboplatin and docetaxel in the first line setting. The study met its primary endpoint showing a superior median PFS of 9.7 months in the erlotinib group compared to 5.2 months in the standard chemotherapy group (HR 0.37; 95% CI 0.25-0.54; p<0.0001). There was no significant difference in overall survival. This is the first randomized trial to demonstrate an improvement of PFS in European patients with EGFR mutation positive NSCLC who received erlotinib compared to standard chemotherapy. (11)

The OPTIMAL study, which was done in 165 Chinese patients, showed that erlotinib is superior to standard chemotherapy in with first line treatment for EGFR mutation positive advanced NSCLC in terms of PFS (HR 0.16, 95% CI 0.10-0.26; p<0.0001). (12)

Afatinib, a second generation TKI that irreversibly inhibits ErbB family tyrosine kinase receptors, showed in two phase 3 randomised studies better PFS in patients with EGFR mutation positive advanced NSCLC receiving afatinib as first line therapy, compared to standard chemotherapy- Lux Lung 3 (HR 0.58; 95% CI, 0.43 to 0.78; P = .001) (13) and Lux Lung 6 (HR 0·28; 95% CI 0·20–0·39; p<0·0001). (14)

The combined data from these studies now define the clinical management of EGFR mutation positive lung cancers. The National Comprehensive Cancer Network (NCCN) and European Society of Medical Oncology (ESMO) currently recommend treatment with a first generation EGFR-TKI (e.g. erlotinib, gefitinib) in the front-line setting for those patients with documented EGFR mutation. Gefitinib is approved in Australia as first line treatment of lung adenocarcinomas with EGFR exon-19 deletion or L858R mutation.

1.3 Acquired resistance to EGFR Tyrosine Kinase Inhibitors

Although first-generation TKIs gefitinib and erlotinib are highly active against EGFR mutation positive advanced NSCLC with objective response rates between 50-70%,(8) the majority of patients will ultimately develop resistance to these agents with progression of disease after 9-13 months of therapy.(6, 15)

Acquired resistance to first generation TKIs can come about through various mechanisms with the most common one due to EGFR T790M ‘gatekeeper’ mutation in approximately 50-60% of cases.(16, 17) The threonine (T) to methionine (M) amino-acid change at position 790 in exon 20 decreases sensitivity of ATP binding-competitive reversible inhibitors and alters binding of the drug to EGFR.(18, 19) It has been originally reported that EGFR-T790M exists as an acquired mutation after exposure to first-generation TKIs; however with more sensitive sequencing technologies it has been revealed that existing EGFR-T790M clones can be detected at low frequencies in patients who are naïve to TKIs. (20) The selection of EGFR-T790M-positive clones under pressure from a first or second generation EGFR TKI may explain the emergence of resistance in this instance highlighting tumour heterogeneity and adaptability. (21)

Other described mechanisms of resistance include the activation of bypass signalling cascades that reactivate downstream MAPK and PI3K pathways, neuroendocrine transformation that silences the expression of or dependence on EGFR protein and c-MET amplification which activates downstream intracellular signalling independent of EGFR in 5-20% of cases.(22-24)

1.4 Treatment of EGFR-T790M positive non-small cell lung cancer

The optimal treatment strategy for patients after failure of first or second-generation EGFR-TKI therapy has previously been limited to chemotherapy. Recent clinical trials, however have indicated significant clinical activity of third generation EGFR TKIs, such as osimertinib, after failure of first-line EGFR TKIs in patients whose tumours harbour T790M mutations.

Second-generation irreversible *EGFR* inhibitors such as afatinib and dacomitinib have shown improvement in PFS in the first-line setting,(25, 26) however, afatinib failed to overcome T790M resistance in patients as monotherapy with a 7% response rate and 2-month improvement in PFS over placebo (median 3.3 versus 1.1 month) in LUX-Lung 1 trial and a similar 8% response rate and 4.4 months median PFS in LUX-Lung 4 trial.(27, 28) Furthermore, concentrations at which these irreversible TKIs overcome T790M activity clinically are not achievable in humans because of dose-limiting toxicity of skin and gastrointestinal effects related to non-selective inhibition of wild-type *EGFR*.(29, 30)

The benefit of cytotoxic chemotherapy in patients with acquired resistance to *EGFR* inhibitors after first-line *EGFR* TKIs are of relatively short duration, with studies showing a median PFS of 4.2-5.5 months.(31, 32)

The third-generation TKIs are designed to target T790M and *EGFR* sensitizing mutations more selectively while sparing the activity of wild-type *EGFR*. Osimertinib (AZD9291; AstraZeneca) is an

oral, potent, irreversible *EGFR* TKI selective for T790M resistance mutation as well as common sensitizing mutations.(29)

1.5 Osimertinib (AZD9291)

The AURA trial tested AZD9291 at doses of 20 to 240mg daily in 253 patients with advanced EGFR mutated NSCLC with radiologically documented disease progression after previous treatment with EGFR-TKI. It included dose escalation cohorts and dose expansion cohorts addressing safety, pharmacokinetics and efficacy of AZD9291. The trial included patients both with, and without T790M resistance mutations.

For all evaluable patients, confirmed RECIST v1.1 responses were observed at all dose levels (20-240mg daily). The overall objective response rate was 51% (95% CI, 45-58%). Among 127 with centrally detectable EGFR-T790M who could be evaluated for response, the response rate was 61% (95% CI, 52-70%) versus 21% (95% CI, 12-34%) in patients without centrally detectable EGFR-T790M. For the 78 patients with centrally tested T790M tumours and confirmed response, the longest duration of response is ongoing at >11 months with a median duration of response at 80mg daily of 8.2 months. The median PFS was 9.6 months (95% CI 8.3 - not reached) in EGFR-T790M positive patients and 2.8 months (95% CI, 2.1-4.3) in EGFR-T790M negative patients.(6)

The phase II AZD9291 open label study (AURA 2) assessed the safety and efficacy of AZD9291 at a dose of 80mg daily in patients with confirmed diagnosis of EGFR and T790M mutation positive NSCLC who have progressed following prior therapy with an EGFR-TKI agent. AZD9291 80 mg once daily demonstrates clinical activity and manageable tolerability in these patients.(33)

AURA 3 is a global, multicentre, phase III, open-label study comparing the efficacy of AZD9291 with platinum-based chemotherapy as second-line treatment in patients following progression of EGFR mutated advanced NSCLC who have received prior EGFR-TKI therapy with the presence of a T790M resistance mutation. This study enrolled chemotherapy naïve patients who were then randomized 2:1 to receive AZD9291 (80mg orally daily) or platinum-based doublet chemotherapy (carboplatin AUC5 or cisplatin 75mg/m2 + pemetrexed 500mg/m2; up to six cycles) according to individual institutional guidelines. The primary objective is to compare the efficacy of AZD9291 versus chemotherapy with the primary endpoint of PFS assessed according to RECIST v1.1. The result of this trial was presented in IASLC 2016 and published recently, showing the median PFS 10.1 months vs. 4.4 months; HR ; 0.30; 95% CI, 0.23 to 0.41; P<0.001. The objective response rate was significantly better with osimertinib (71%; 95% CI, 65% to 76%) than with platinum therapy plus pemetrexed (31%; 95% CI, 24% to 40%) (odds ratio for objective response, 5.39; 95% CI, 3.47 to 8.48; P<0.001).(34) Among the 144 patients with CNS disease at baseline, the median PFS was 8.5 months vs. 4.2 months; HR 0.32; 95% CI, 0.21 to 0.49).

Osimertinib has been granted FDA approval in the United States and has received approval in the European Union, Japan and Australia based on the above data..

1.6 EGFR T790M mutation - plasma cfDNA testing

With the regulatory approval of osimertinib in the United States, Europe, Japan and Korea for treatment of metastatic EGFR-T790M NSCLC, this mutation now acts as a biomarker for guidance of treatment in patients with acquired resistance to prior EGFR-TKI.(35) Tumour genotyping for EGFR-T790M requires a biopsy. However, the acquisition of tissue continues to be a challenge with both the INTEREST and IPASS studies reporting only 31-42% of patients had tissue available or tissue that could be obtained for molecular testing.(8, 36) Moreover, detection of relevant mutation from a biopsy of a single tumour lesion may not reflect the patient's complete disease burden.

In the last 5 years, the detection and sequencing of tumour-derived cell free DNA (cfDNA) in plasma by so-called "liquid biopsy" has enabled serial, non-invasive assessment of patients' mutation status with the added advantage of decreasing sampling bias due to intra-tumoural heterogeneity. It has been shown in several lung cancer studies that EGFR mutation detected in plasma are highly concordant with those detected in tumour tissue indicating that cfDNA as liquid biopsy is feasible and can be an alternative to tissue biopsy.(35, 37) The measurement of plasma

cfDNA provides a unique opportunity to personalise treatment decisions by serially monitoring patient specific cancer mutations in real-time using a minimally invasive technique.

Several technologies have been used to analyse plasma cfDNA EGFR mutations such as the cobas® EGFR Mutation Test and BEAMing dPCR with a high sensitivity of 82-86% for sensitizing mutations and 70% for EGFR-T790M mutation. Plasma genotyping is also very specific for the sensitizing mutations with specificity of 98% but is lower for the EGFR- T790M mutation with specificity of 69%.(35)

It has been shown that this non-invasive method of using plasma cfDNA as an assay to detect EGFR-T790M mutation is highly sensitive (38). This was a retrospective study where plasma was collected from all patients in the first-in-man study of osimertinib. These patients had acquired EGFR-TKI resistance and evidence of a common EGFR-sensitizing mutation. Sensitivity of plasma genotyping for detection of EGFR-T790M was 70%. Patients with positive EGFR-T790M mutation in plasma have outcomes with osimertinib that are equivalent to patients positive by a tissue-based assay. (38)

1.7 Rationale for this study

Several different mechanisms of resistance have been described including acquired C797S mutations and loss of EGFR-T790M, both of which confer resistance to the third generation EGFR-TKI osimertinib. The characterisation of mechanism of resistance in 22 patients who became resistant to AZD9291 was reported from the AURA I/II trial. Cell-free DNA (cfDNA) from the plasma of these patients were analysed by next-generation sequencing (NGS) at disease progression. Plasma C797S was detected in 40% of patients with detectable plasma EGFR-T790M concentrations, all harbouring an exon 19 deletion as their EGFR activating mutation. In 33% of patients, the EGFR-T790M mutation was detected at progression without evidence of the C797S mutation and in another 27%, the EGFR-T790M mutation was no longer detectable at progression despite detectable EGFR-T790M cfDNA before AZD9291 treatment. (39)

These findings have demonstrated genomic heterogeneity associated with resistance to EGFR TKIs in NSCLC, which requires further study and novel strategies to prevent or delay mechanisms of resistance to osimertinib. This indicates a need for combination therapies that are likely to enhance the durability of response and clinical benefit seen in patients with EGFR-T790M positive NSCLC treated with osimertinib.

The changes in clonal predominance upon long-term exposure or adaptation of the tumour mass to first, second and ultimately third generation EGFR-TKIs forms the rationale of alternating treatment. Laboratory derived cell lines from biopsies of cancers that have become resistant to targeted therapies have shown that the C797S mutation in the absence of EGFR-T790M is sufficient to cause resistance to third generation TKIs but retains sensitivity to gefitinib which raises the possibility that patients with acquired resistance to third generation TKI may subsequently respond to first or second generation TKI. (40)

The Oscillate study is expected to provide new insights into the efficacy of alternating osimertinib and gefitinib and its impact on the dynamics of plasma EGFR-T790M cfDNA levels.

An initial period of “induction osimertinib” is proposed to achieve maximum or near maximal cytoreduction prior to introducing the alternating gefitinib and osimertinib. It is expected that the proportion of patients with early progression will be less with this approach, compared with starting with the alternating strategy upfront.

The concept of tumour evolution and the contribution of clonal heterogeneity to the development of resistance to therapy with disease progression therapy has been established in many cancers including NSCLC. It is likely that subclones of EGFR-T790M mutated cancer cells are present at low frequency when treatment with a first generation EGFR TKIs is started and that treatment with the first-generation EGFR TKI will apply a selective pressure that increases the proportion of these EGFR-T790M mutated cells until they become the dominant clone and become evident at tumour progression. Paradoxically, in preclinical studies EGFR-T790M+ clones are said to have a more indolent growth when compared with the parental cell lines that they are derived from. Furthermore, patients with resistance due to EGFR-T790M mutations appear to have better

prognosis than patients without EGFR T790M mutations. It may therefore be possible to exploit this clonal heterogeneity for therapeutic advantage by maintaining a dynamic equilibrium between T790M+ cells and EGFR-T790M- cells to prevent emergence of alternative mechanisms of resistance less amenable to therapeutic manipulation.

We hypothesise that alternating therapy with osimertinib and the first generation EGFR TKI gefitinib will modulate the population of clones within the tumour by maintaining a dynamic balance between EGFR-T790M positive and negative tumour clones, delaying the emergence of resistance to osimertinib.

2 AIM AND OBJECTIVES

**Aims**	To determine the efficacy, safety, and feasibility of alternating osimertinib and gefitinib in patients with EGFR-T790M mutation positive NSCLC.
**Objectives**	To determine the
**Primary**	1. PFS rate at 12 months according to RECIST v1.1
**Secondary**	2. feasibility of alternating osimertinib and gefitinib
	3. time to progression (TTP) and PFS time
	4. objective tumour response rate (OTRR=CR+PR, RECIST 1.1)
	5. overall survival (OS)
	6. frequency and severity of adverse events (CTCAE v4.03)
**Tertiary correlative**	7. changes in plasma cfDNA levels for activating EGFR mutations and EGFR-T790M mutation over time
	8. relationships between changes in plasma cfDNA levels for activating EGFR mutations, EGFR-T790M mutation, and OTRR
	9. mechanisms of resistance in patients progressing on alternating osimertinib and gefitinib as identified in cfDNA (or where possible tumour biopsy)

3 DESIGN

This is an open-label, single arm, multi-centre, phase 2 trial with a safety run in.

4 STUDY POPULATION

Patients must meet all of the inclusion criteria and none of the exclusion criteria to be eligible for this trial. No exceptions will be made to these eligibility requirements at the time of registration. All enquiries about eligibility should be addressed by contacting the NHMRC CTC prior to registration.

4.1 Target Population

Adults with advanced, EGFR mutated NSCLC that have acquired resistance to first or second generation EGFR-TKIs and are EGFR-T790M mutation positive.

4.2 Inclusion Criteria

1. Adults, aged 18 years and older, with histologically or cytologically confirmed metastatic or unresectable locally advanced NSCLC
2. Prior therapy with an EGFR-TKI. Patients may also have received additional lines of treatment
3. Documented evidence of EGFR-T790M mutation on tissue and/or plasma sample following disease progression on the most recent EGFR-TKI therapy (T790M mutation status will

need to be re-confirmed in the event of an alternative systemic treatment following progression on the most-recent EGFR-TKI therapy).

4. Measurable disease according to RECIST version 1.1. Previously irradiated lesions can only be considered as measurable disease if disease progression has been unequivocally documented at that site since radiation, and the previously irradiated lesion is not the only site of disease
5. Eastern Cooperative Oncology Group (ECOG) performance status 0-2
6. Adequate bone marrow function (within 28 days prior to registration and with values within the ranges specified below):
   - Absolute neutrophil count (ANC) ≥ 1.5 x 10^9/L
   - Platelets >100 x 10^9/L
   - Haemoglobin ≥ 90 g/L
7. Adequate liver function (within 28 days prior to registration and with values within the ranges specified below):
   - Aspartate aminotransferase (AST) and alanine aminotransferase (ALT) ≤ 3 x upper limit of normal (ULN); if liver metastases ≤ 5 x ULN
   - Bilirubin ≤ 2 x ULN
8. Adequate renal function (within 28 days prior to registration and with values within the ranges specified below):
   - Serum creatinine ≤ 1.5 x ULN

     or
   - Creatinine clearance (CrCl) ≥ 50 mL/min (use Cockroft-Gault formula as per section 17.1, appendix 1)
9. Willing and able to comply with all study requirements, including treatment , timing and/or nature of required assessments
10. Women must be post-menopausal, infertile, or use a reliable means of contraception. Women of childbearing potential must have a negative serum pregnancy test done within 24 hours prior to enrolment. Men must have been surgically sterilised or use a barrier method of contraception (condoms) if they are sexually active with a woman of child bearing potential. See section 5.5.6 for further details.
11. Signed, written informed consent (main study and tissue banking).

4.3 Exclusion Criteria

1. Previous or current treatment with osimertinib or other drugs that target EGFR-T790M mutations, e.g. CO-1686, HM61713, TAS-121
2. Contraindications to investigational product
3. Any unresolved toxicity from prior therapy worse than CTCAE grade 1, except alopecia and grade 2 neuropathy due to prior platinum-based chemotherapy
4. Major surgery within 4 weeks, or palliative radiation therapy within 5 days before registration
5. Treatment with prohibited medications (e.g. concurrent anti-cancer therapy including other chemotherapy, or immunotherapy ≤ 14 days prior to treatment)
6. Patients currently receiving (or unable to stop at least 1 week before starting osimertinib) potent inhibitors or inducers of cytochrome P450 (CYP) 3A4 (see table 2 of Appendix 2)

7. Patient with symptomatic central nervous system (CNS) metastases who are neurologically unstable, or require increasing doses of steroids to manage CNS symptoms within 2 weeks prior to starting osimertinib. Patients with leptomeningeal carcinomatosis are also excluded
8. Known history of interstitial lung disease from any cause
9. Life expectancy of less than 3 months
10. Mean QT interval corrected for heart rate (QTc) ≥ 470 ms calculated from 3 baseline ECGs using Fridericia's Correction ($QTc = QT/RR^{1/3}$) OR any clinically important abnormalities in rhythm, conduction or morphology of resting ECG (e.g. complete left bundle branch block, third degree heart block, second degree heart block) OR any factors that increases the risk of QTc prolongation or risk of arrhythmic events
11. History of another malignancy within 5 years prior to registration. Patients with a past history of adequately treated carcinoma-in-situ, basal cell carcinoma of the skin, squamous cell carcinoma of the skin, or superficial transitional cell carcinoma of the bladder are eligible. Patients with a history of other malignancies are eligible if they have been continuously disease free for at least 5 years after definitive primary treatment
12. Any evidence of severe or uncontrolled systemic diseases, including uncontrolled hypertension, active bleeding diatheses, active infection including hepatitis B, hepatitis C and human immunodeficiency virus (HIV), or significantly impaired bone marrow reserve or organ function, including hepatic and renal impairment, which in the investigator's opinion would significantly alter the risk/benefit balance
13. Any condition that, in the opinion of the investigator, would interfere with evaluation of study treatment or interpretation of patient safety or study results
14. Serious medical or psychiatric conditions that might limit the ability of the patient to comply with the protocol.

4.4 Study Enrolment

4.4.1 Screening

Written informed consent must be signed and dated by the participant, and signed and dated by the Investigator, prior to any study-specific screening investigations being performed.

4.4.2 Registration

Registration will be done according to the instructions in the Study Manual. Once the registration process has been completed, the participant will be assigned a participant study number. Individuals may only be registered once in this trial.

Written confirmation of registration will be provided to the site.

Requests for registration will only be accepted from authorised investigators at sites that have all requisite approvals in place. Registration should be done only after all screening assessments have been performed and the responsible investigator has verified the participant's eligibility.

Study treatment must be planned to start within ***7*** days of registration.

5 TREATMENT PLAN

5.1 Treatments

All participants will receive:

Phase	Drug	Dose	Route	Duration
**Induction**	Osimertinib	80mg daily	PO	8 weeks (2 x 4 weekly cycles)
**Alternating**	Gefitinib	250mg daily	PO	Alternating 4 week cycles of each drug, i.e. gefitinib for 4 weeks, then osimertinib for 4 weeks, then gefitinib for 4 weeks, etc) until disease progression or prohibitive toxicity.
	Alternating with			
	Osimertinib	80mg daily	PO	
**Post-progression***	Osimertinib	80mg daily	PO	Until further progression or prohibitive toxicity

*Continuous dosing with osimertinib after progression on alternating therapy may be permitted after consultation with delegates of the Trial Executive Committee if the subject meets the criteria listed in section 5.8.

5.1.1 Required Background Treatment

There is no required background medication or premedication for osimertinib and gefitinib.

5.2 Administration of treatment

Osimertinib (previously known as AZD9291) will be supplied as tablets for oral administration as a single daily dose of 80mg and supplied in blister packs of 30 tablets. Osimertinib 40mg tablets will be supplied for use for dose reductions as per protocol.

Gefitinib will be supplied as tablets for oral administration as a single daily dose of 250mg daily. Each bottle will contain sufficient gefitinib treatment for 28 days.

Doses should be taken approximately 24 hours apart at the same time point each day. Doses should not be missed. If a patient misses taking a schedule dose, within a window of 12 hours, it is acceptable to take the dose. If it is more than 12 hours after the schedule dose time, the missed dose must not be taken, and the patients must be instructed to take the next dose at the next scheduled time. If a patient vomits after taking their study treatment, they must not make up for this dose, but must take the next scheduled dose.

In addition to the blood tests on day 15 of cycle 3 and 4 (the first 2 cycles involving a switch in therapy from osimertinib to gefitinib and gefitinib to osimertinib), the first 6 participants will have a clinical review to identify potential toxicities related to immediately switching between TKIs. Delegates of the TMC will review the blood results and toxicity on an ongoing basis until the 6th participant has completed 4 cycles of treatment. If no safety signal of concern is seen at this point, the additional clinical reviews on day 15 of cycles 3 and 4 will stop, but the blood tests will continue to be taken for subsequent participants without the need for a clinical review. There is no plan to suspend accrual during this process unless there is a safety signal.

5.3 Dose Modifications

The initial dose of osimertinib 80mg daily can be reduced to 40mg once daily under circumstances described in Section 5.3.1.

The initial dose of gefitinib is 250mg daily with no dose reductions.

Any change from dosing schedule or dose interruptions must be recorded in the (e)CRF. Please note for osimertinib only dose reductions must be recorded in the (e)CRF.

5.3.1 Dose Modifications for Adverse Events

If a patient experiences a CTCAE ≥ grade 3 and/or an unacceptable toxicity of any grade, where the investigator considers the event of concern to be specifically associated with the trial medications (and not attributable to the disease or disease-related process for which patient is being treated), dosing will interrupted and supportive therapy administered as required in accordance with local practice/guidelines. If the toxicity resolves or reverts to CTCAE grade ≤ 1 within 3 weeks of onset, then that study drug may be later restarted, when due, at either the same dose (e.g. osimertinib 80 mg daily, gefitinib 250 mg) or a lower dose (osimertinib 40mg daily) using the rules below in Table 1, as per the investigator's evaluation and discussion with delegates of the TMC.

Once a dose reduction of osimertinib is implemented, the dose of osimertinib cannot be re-escalated to 80mg.

There are no dose reductions for gefitinib, only dose interruptions.

The dates of the cycles will remain as scheduled and will not be delayed i.e. if a dose interruption occurs on day 22 of a gefitinib cycle and the patient is able to resume therapy on day 8 of the following cycle the patient will resume treatment with osimertinib from day 8 and continue it to day 28.

If a treatment-related adverse event does not resolve to CTCAE grade 0-2 within 3 weeks, then permanent discontinuation from study treatment should be considered after discussion with delegates of the TMC. Participants should be followed until the resolution of toxicity due to study treatment.

Table 1: Management of osimertinib and gefitinib Treatment-Related Toxicity

Toxicity	NCI CTCAE Severity Grade	Osimertinib	Gefitinib
		**Treatment Modification**	**Treatment Modification**
Liver function Abnormalities	Grade 1<br>AST or ALT > ULN to 3.0 x ULN and/or total bilirubin > ULN to 1.5 x ULN	Continue on osimertinib	Continue on gefitinib
	Grade 2<br>AST or ALT > 3.0 to ≤ 5.0 x ULN and/or total bilirubin >1.5 to ≤ 3.0 ULN	Withhold osimertinib until resolves ≤ Grade 1, then resume at same dose level.	Withhold gefitinib until resolves ≤ Grade 1, then resume at same dose level
	Grade 3 or 4:<br>AST or ALT >5.0 x ULN and/or total bilirubin >3 x ULN	Withhold osimertinib until resolves ≤ Grade 1, then resume at reduced dose of 40mg daily	Withhold gefitinib until resolves ≤ Grade 1, then resume at same dose level
Skin reactions<br>Rash treatment	Grade 1:<br><10% body surface area (BSA) papules/pustules<br>With or without symptoms of pruritus or tenderness	Continue on osimertinib Emollient cream application and/or<br>Topical steroid moderate strength BD and/or<br>Topical antibiotic BD	Continue on gefitinib<br>Emollient cream application and/or<br>Topical steroid moderate strength BD and/or<br>Topical antibiotic BD
	Grade 2:<br>10-30% BSA papules/pustules with or without symptoms of pruritus or tenderness<br>Psychosocial impact<br>Limiting ADLs	Continue on osimertinib Treatment same as for Grade 1<br>Consider using oral antibiotic for 6 weeks	Continue on gefitinib<br>Treatment same as for Grade 1<br>Consider using oral antibiotic for 6 weeks
Skin reactions<br>Rash treatment	≥ Grade 3:<br>>30% BSA papules/pustules with or without symptoms of pruritus or tenderness<br>Limiting self-care ADLs<br>Associated with local superinfection	Topical steroid moderate strength BD and oral antibiotics for 6 weeks<br>Switch to broad-spectrum antibiotics if infection suspected.<br>Consider skin swab for bacterial culture.<br>Consider withholding for up to 3 weeks until settled to ≤ Grade 1.	Topical steroid moderate strength BD and oral antibiotics for 6 weeks<br>Switch to broad-spectrum antibiotics if infection suspected.<br>Consider skin swab for bacterial culture.<br>Consider withholding for up to 3 weeks until settled to ≤ Grade 1.

Toxicity	NCI CTCAE Severity Grade	Osimertinib Treatment Modification	Gefitinib Treatment Modification
Diarrhoea	Grade 1-2	Continue on osimertinib<br>Grade 1 intermittent diarrhoea may not require treatment.<br>Loperamide: 4mg, followed by 2mg every 4 hours or after every unformed stool<br>Consider continuation of loperamide until diarrhoea free for 12 hours<br>Consider electrolyte replacement	Continue on gefitinib<br>Grade 1 intermittent diarrhoea may not require treatment.<br>Loperamide: 4mg, followed by 2mg every 4 hours or after every unformed stool<br>Consider continuation of loperamide until diarrhoea free for 12 hours<br>Consider electrolyte replacement
	Grade 3-4	Consider holding drug for up to 3 weeks.<br>If dehydration is severe, use intravenous fluid and consider octreotide.<br>Stool culture.<br>Consider prophylactic antibiotics, especially if diarrhoea is persistent beyond 24 hours or there is fever or Grade 3-4 neutropenia<br>Electrolyte replacement as appropriate	Consider holding drug for up to 3 weeks.<br>If dehydration is severe, use intravenous fluid and consider octreotide.<br>Stool culture.<br>Consider prophylactic antibiotics, especially if diarrhoea is persistent beyond 24 hours or there is fever or Grade 3-4 neutropenia<br>Electrolyte replacement as appropriate

Toxicity	NCI CTCAE Severity Grade	Osimertinib	Gefitinib
		**Treatment Modification**	**Treatment Modification**
QTc prolongation	Grade 1:<br>QTc 450-480ms	Continue osimertinib	Continue gefitinib
	Grade 2:<br>QTc 481-500ms	Continue osimertinib at same dose level. Assess electrolytes and concomitant medication. Correct any electrolyte or magnesium abnormalities.	Continue gefitinib at same dose level. Assess electrolytes and concomitant medication. Correct any electrolyte or magnesium abnormalities.
	Grade 3 confirmed as >500ms absolute or >60ms increase from baseline	Interrupt dose and perform regular ECG until resolution to baseline.<br>If QTc resolves to Grade 1, re-start osimertinib at 40mg daily.<br>If QTc does not resolve to ≤Grade 1 (<481ms) after 3 weeks, then permanently discontinue osimertinib and observe until resolution of toxicity.	Interrupt dose and perform regular ECG until resolution to baseline.<br>If QTc resolves to Grade 1, re-start gefitinib at same dose.<br>In case of recurrence, withhold until recovery to Grade ≤ 1, then re-start at same dose.<br>Permanently discontinue if further ≥ 3 recurrence.
	Grade 4 QTc interval prolongation and/or signs/symptoms of serious arrhythmia	Permanently discontinue osimertinib	Permanently discontinue gefitinib
Ocular disorders<br>Keratitis, corneal erosion, aberrant eyelash growth, conjunctivitis, blepheritis and dry eye	Grade 2	Consider withholding osimertinib for up to 3 weeks.<br><br>Consult with ophthalmologist and perform visual slit lamp test.<br><br>Re-start osimertinib at 80mg or reduced dose of 40mg daily.	Consider withholding gefitinib for up to 3 weeks.<br><br>Re-start gefitinib at same dose.
	Grade 3 or 4	Withhold osimertinib for up to 3 weeks. If resolved to ≤ Grade 1, to resume at 40mg daily.<br>If not improving in 3 weeks, consider permanent discontinuation.	Withhold gefitinib for up to 3 weeks. If resolved to ≤ Grade 1, to resume at same dose.<br>If not improving in 3 weeks, consider permanent discontinuation.

Toxicity	NCI CTCAE Severity Grade	Osimertinib<br>Treatment Modification	Gefitinib<br>Treatment Modification
Suspected Interstitial Lung Disease (ILD)<br>Cough/ dyspnea/ radiological abnormality	Any grade	Withhold osimertinib	Withhold gefitinib
		Perform full diagnostic workup, to exclude alternative causes such as lymphangitis carcinomatosis, infection, allergy, cardiogenic edema, or pulmonary haemorrhage.<br>The results of the full diagnostic workup (including high-resolution computed tomography, blood and sputum culture, haematological parameters) will be recorded in the CRF by the investigator in the event of an SAE.<br>Where ILD is suspected, local practice must be followed in discussion with delegates of TMC. In the absence of a confirmed diagnosis of ILD, osimertinib and/or gefitinib may be restarted following consultation with delegates of the TMC.<br>In the case of a confirmed diagnosis of ILD, osimertinib and gefitinib must be permanently discontinued.	

Patients experiencing corneal ulceration, Interstitial Lung Disease (ILD) or QTc prolongation with signs/symptoms of serious arrhythmia will **not be permitted to restart study treatment**.

5.4 Post-study treatment

Treatment after discontinuation of study treatment is at the discretion of the patient's clinician. If the patient is still benefiting from study treatment at the end of the study, ongoing supply will be provided by the manufacturer until the product is available to the public.

Please refer to section 5.8 for treatment beyond progression.

5.5 Concomitant Medications

Details of medications that may interact with study treatment are listed in Appendix 2.

5.5.1 Anticancer or experimental, radiotherapy or surgical therapy

No other systemic anti-cancer therapies (including chemotherapy, hormonal treatment [except corticosteroids and megestrol acetate], antibody or other immunotherapy, or other experimental drugs) of any kind will be permitted while the patient is participating in the study.

Palliative radiotherapy or local surgical treatment to specific sites of disease is permitted if considered medically necessary by the treating physician and there is no disease progression. All attempts should be made to rule out disease progression in the event of increased localised pain. If the palliative radiotherapy is needed to control bone pain, the sites of bone disease should be present at baseline, otherwise, bone pain requiring radiotherapy will be considered as a sign of disease progression. All procedures performed (e.g. radiotherapy, surgery, thoracentesis, etc.) undertaken during the study must be documented in the patient's medical record.

Osimertinib or gefitinib must be stopped 3-5 days before and after completion of any radiation/surgical therapy.

5.5.2 Hematopoietic Growth Factors and Blood Products

Erythropoietin, darbepoetin alfa, and/or hematopoietic colony-stimulating factors for treatment of cytopenias should be administered according to institutional guidelines. Prophylactic use of these agents is not permitted.

Transfusion thresholds for blood product support will be in accordance with institutional guidelines.

5.5.3 Other Concomitant Medications

Therapies considered necessary for patients' well-being may be given at the discretion of the investigator. Other concomitant medications, except for analgesics, chronic treatments for concomitant medical conditions, or agents required for life-threatening medical problems, should be avoided. Herbal and complementary therapies should not be encouraged because of unknown side effects and potential drug interactions.

5.5.4 Contra-indicated medications

Strong inducers of CYP3A4 can significantly decrease both osimertinib, and gefitinib exposure, compromising efficacy. Potent inhibitors can significantly increase the gefitinib exposure raising the risk of toxicity. See Appendix 2.

5.5.5 Use with Caution

The following medications **should be avoided if possible** whilst participants are on study drug. If there are no alternatives, these drugs **may be continued/used with caution**:

- Medications known to produce QT prolongation (See Appendix 2). The use of such concomitant medications and an appropriate ECG monitoring plan should be agreed between the CTC and Study Chair. Acceptable anti-emetics with low potential to affect QTc include phenothiazines and corticosteroids.
- Drugs affecting gastric pH, as pH >5.0 led to a significant reduction in gefitinib exposure
- CYP450 inducers and inhibitors can affect osimertinib and gefitinib exposure, leading to under-dosing or over-dosing and should be used with caution. Appendix 2 provides a more extensive list of medications to be avoided, and includes medications that are known to do the following;
    - Drugs that are sensitive substrates of CYP3A4, breast cancer resistance protein (BCRP), or CYP1A2 with narrow therapeutic indices
    - Strong inhibitors of CYP2C8 and CYP2D6, which can decrease osimertinib exposure
    - Drugs known to inhibit CYP2C8, CYP2C9 and CYP2C19, as osimertinib moderately inhibits these enzymes

5.5.6 Guidance regarding potential interactions with specific concomitant medications/devices

- Statins
    - Up to 3-fold increase in exposure may occur in statin exposure when co-administered with osimertinib. It is recommended that the starting and maintenance dose of statins should be as low as possible and should be guided by the statin prescribing information.

- Warfarin/Coumadin
    - Patients taking warfarin should be monitored regularly for changes in prothrombin time or international normalized ratio.

- Contact lenses
    - Patients who wear contact lenses must discontinue wearing their lenses if they have any mild to moderate eye symptoms (CTCAE grade ≤ 2) while receiving treatment with osimertinib until at least one week after symptoms have resolved. If a patient has a recurrence of eye symptoms or experiences any severe (CTCAE grade ≥ 3) ocular events, they must discontinue wearing their contact lenses until at least one week after treatment with osimertinib is permanently discontinued. Patients must not use any eye drops or ointment for treatment of eye symptoms, unless agreed to by a study doctor, at any time during the study until 1 week after osimertinib has been permanently discontinued. Patients must consult their investigator promptly if they have any concerns.

5.5.7 Concomitant Medication Reporting

Concomitant medications are only recorded in the event of a serious adverse event report. Concomitant medications used to treat an SAE will not be recorded.

5.5.8 Restrictions for sexually active participants

5.5.8.1 Females of child-bearing potential

Females of child-bearing potential should use reliable methods of contraception from the time of screening until 6 weeks after discontinuing study treatment. Acceptable methods of contraception include total and true sexual abstinence, tubal ligation, hormonal contraceptives that are not prone to drug-drug interactions (e.g. IUS Levonorgestrel Intra Uterine System (Mirena), medroxyprogesterone injections (Depo-Provera)), copper-banded intra-uterine devices and vasectomised partner. All hormonal methods of contraception should be used in combination with the use of a condom by their male sexual partner for intercourse.

5.5.8.2 Male participants

Male participants should be asked to use barrier contraceptives (i.e. by use of condoms) during sex with all partners during the trial and for a washout period of 4 months. Subjects should not father a child for 4 months after completion of study treatment. Subjects should refrain from donating sperm from the start of dosing until 4 months after discontinuing study treatment. If male subjects wish to father children they should be advised to arrange for freezing of sperm samples prior to the start of study treatment.

5.6 Compliance

Patient treatment compliance will be determined by their history during their follow up clinic for study treatment assessment. Patients must return unused tablets/empties of their study treatment(s) at each visit. The study team will review the number of returns and counsel the patient appropriately if significant non-compliance is determined.

5.7 Study Treatment Discontinuation

Study treatment must be permanently discontinued for any of the following reasons:

- Unacceptable toxicity as determined by the patient or site investigator or as defined in section 5.3
- Drug-induced corneal ulceration
- Confirmed diagnosis of interstitial lung disease (ILD)
- QTc prolongation with symptoms/signs of serious arrhythmia
- If gefitinib is discontinued permanently because of tolerability concerns, the patient may continue with osimertinib if agreed upon by the investigator, patient and the sponsor.

- If osimertinib is discontinued permanently because of tolerability concerns, the patient will come off study.
- Occurrence of an exclusion criterion affecting patient safety, e.g. pregnancy or psychiatric illness
- Required use of a concomitant treatment that is not permitted, as defined in section 5.5
- Failure to comply with the protocol, e.g. repeatedly failing to attend scheduled assessments
- Initiation of alternative anticancer therapy including another investigational agent
- The patient declines further study treatment, or withdraws their consent to participate in the study.

The reasons for discontinuing treatment will be documented in the participant's medical record and (e)CRF.

Participants who stop study treatment prior to the time recommended in the protocol will be requested to continue follow-up visits according to the protocol.

If a participant wishes to stop the study visits, they will be requested to allow their ongoing health status to be periodically reviewed via phone contact or from their general practitioner, or medical records.

5.8 Treatment beyond progression

Patients may be considered for treatment with continuous osimertinib beyond radiographic progression per RECIST v1.1 at the discretion of the investigator and after appropriate discussion with the patient and if the following criteria are met:

- Evidence of clinical benefit as assessed by the investigator
- Absence of symptoms and signs indicating unequivocal progression of disease
- No decline in ECOG performance status that can be attributed to disease progression
- Absence of tumour progression of critical anatomical site (i.e. leptomeningeal disease) that cannot be managed by protocol-allowed medical interventions
- The decision to defer alternative treatment options in favour of continuing study treatment at the time of initial progression is documented in the patient's medical record, and in the Case Report Form as A Note To File.

6 ASSESSMENT PLAN

6.1 Schedule of Assessments

	Screening	On study treatment				End of alternating treatment/ withdrawal	After alternating study treatment	
		Cycles 1-2 Osimertinib	Cycle 3 Gefitinib	Cycle 4 Osimertinib	Cycle ≥ 5 Gefitinib alternate with osimertinib		Until progression/ withdrawal on osimertinib alone	After progression
		Induction phase	Alternating phase					
	Within 28 days before registration	Each cycle is 28 days Day 1 (+/- 3 days)				28 days +/- 7 days after last dose	Every 4 weeks +/- 7 days	Every 12 weeks +/- 7 days
Informed consent	X							
Medical history	X							
Clinical assessment [1]	X	Day 1 of each cycle, plus day 15 of cycles 3 and 4 [2]				X	X	
Vital signs and weight	X	X				X		
ECOG performance status	X							
Confirm EGFR-T790M mutation on tissue and/or plasma	X							
Blood for haematology/biochemistry [3]	X	Day 1 of each cycle, plus day 15 of cycles 3 and 4 [4]				X	X	
Pregnancy test in WOCBP [5]	X							
Urinalysis [6]	X							
12-lead ECG	X	X [7]				X [7]		
CT chest, abdomen and pelvis [8]	X	Every 8 weeks (2 cycles)					X[8]	
Contrast enhanced CT or MRI brain [9]	X	Every 8 weeks (2 cycles) if intracranial disease present at baseline					X[9]	
Concomitant medications [10]	X	X[10]						
LVEF by GHBPS (MUGA) or ECHO [11]	X	Every 16 weeks (4 cycles) whilst on study treatment [11]					X[11]	
Ophthalmologic assessment [12]	X	If ocular symptoms develop whilst on study treatment [12]					X[12]	
Adverse Events		X[13]				X[13]	X[13]	
Blood for EGFRT790M mutation testing and translational research[14]	X	Day 1 of each cycle, plus day 15 of cycles 3 and 4 [14]				X[14]		
Survival and anticancer treatment[15]								X[15]
Biopsy or pleural effusion sample for research [16] (optional)						X[16]		

Footnotes

1. Directed history, physical examination, and vital signs as per standard of care (not recorded in CRF). Does not need to be repeated if done within the last 7 days
2. **An extra clinical review will be scheduled on day 15 of cycles 3 and 4 to review toxicity following the switch from osimertinib to gefitinib and from gefitinib to osimertinib. Following review of toxicity from the first 6 participants, the requirement for this visit may be removed.**
3. Samples for the following laboratory tests will be sent to the study sites local laboratory for analysis:
   Haematology tests must include a full blood count and differentials. Biochemistry tests must include the following: glucose, urea, creatinine, sodium, potassium, magnesium, chloride, bicarbonate, calcium, phosphorus, total bilirubin, ALT, AST, alkaline phosphatase, LDH, total protein and albumin.
4. An **extra** set of blood tests for haematology and biochemistry (including liver function tests) as well as plasma cfDNA is required at **day 15 of cycles 3 and 4.**
5. Serum or urine BHCG for women of child-bearing potential (WOCBP) must be tested.
6. Urinalysis (specific gravity, pH, glucose, protein, ketones, and blood); dipstick permitted.
7. As clinically indicated.
8. CT chest, abdomen and pelvis must be done at baseline, week 8 and 8 weekly thereafter (2 cycles) during study treatment (including select cases continued beyond progression with single-agent osimertinib).
9. CT or MRI brain is indicated for assessment at baseline. Brain must be included in subsequent tumour assessments if a patient has brain metastases (including select cases continued beyond progression with single-agent osimertinib).
10. Concomitant medications should be recorded in the hospital medical record at each visit until 30 days after the last dose of study treatment (including select cases continued beyond progression with single-agent osimertinib), but are **only** recorded in the CRF when reporting SAE.
11. An Echocardiogram or MUGA scan to assess LVEF will be performed at screening (prior to first dose of osimertinib) and at least every 16 weeks throughout the treatment period. The modality of the cardiac function assessments must be consistent within a patient i.e. if echocardiogram is used for the screening assessment then echocardiogram should also be used for subsequent scans. The patients should also be examined using the same machine and operator whenever possible, and quantitative measurements should be taken. A 28-day follow-up assessment will be required if an on treatment assessment was abnormal at the time of discontinuation of study therapy, to confirm reversibility of the abnormality.
12. Full ophthalmic assessment, including slit lamp examination, will be performed at screening and should be repeated if a patient experiences any visual symptoms (including blurring of vision), with additional tests if clinically indicated. Any clinically significant findings, including those confirmed by the ophthalmologist must be reported as an AE. Photographs should be performed to record any clinically significant findings. Any patient developing corneal ulceration will be permanently discontinued from study treatment and should be followed regularly until resolution of the event.
13. Adverse events collected up to 30 days after last dose of study treatment.
14. Refer to Biospecimen Sampling Manual for details of collection, processing and storage.
15. Contact by phone is acceptable.
16. At the time of disease progression.

6.2 Details of Assessments

6.2.1 Screening phase

Written informed consent must be signed and dated by the participant, and signed and dated by the Investigator, prior to any study-specific screening investigations being performed.

6.2.2 Treatment Phase

Procedures to be conducted during the treatment phase of the study are presented in section 6.1 schedule of assessments. All participants must commence treatment within *7* days of registration.

6.2.3 Clinical assessment

6.2.3.1 Medical history

Medical history includes significant diseases, surgeries, cancer history (including prior cancer therapies and procedures), reproductive status, smoking history, and all medications (e.g. prescription drugs, over-the-counter drugs, herbal or homeopathy remedies, nutritional supplements) used by the patients within 14 days prior to the screening visit.

6.2.3.2 Physical examination

A complete physical examination should include an evaluation of the respiratory, cardiovascular, gastrointestinal, neurological and musculoskeletal systems. A targeted physical examination as clinically indicated will be performed on subsequent evaluations.

6.2.3.3 Vital signs

Vitals signs (blood pressure, pulse rate, respiratory rate and temperature) will be measured at baseline and on subsequent clinical assessment days as scheduled in section 6.1. These assessments are not recorded in the eCRF.

6.2.3.4 Electrocardiogram (ECG)

ECGs are required during screening, as well as at any other time point when clinically indicated. ECGs recorded during the screening period will be obtained in triplicate (with 2-5 minute lag time between each). ECGs recorded during the treatment phase if clinically indicated will be single tracing.

All 12-lead ECGs should be recorded while the participant is in the supine position. A 12-lead ECG will be recorded for all participants as noted in section 6.1 schedule of assessments. If additional ECGs are required the same method of assessment should be used throughout the study. Twelve-lead ECGs will be obtained after the participant has been resting in a supine position for at least 5 minutes in each case.

6.2.4 Imaging

Screening assessments must include CT scans (with oral/IV contrast unless contraindicated) of the chest, abdomen and pelvis (staging). Subsequent staging CTs should be done at week 8 and 8-weekly thereafter (every 2 cycles of study treatment) during study treatment.

A CT brain (with contrast if not contraindicated) must be performed at screening to exclude CNS metastasis. An MRI scan of the brain is required to confirm or refute the diagnosis of CNS metastases at baseline in the event of an equivocal scan. Patients with asymptomatic or stable brain metastases will require repeat brain imaging as part of follow-up tumour assessments, with MRI brain as the preferred modality on study.

Tumour assessments performed as standard of care prior to obtaining informed consent and within 28 days of Cycle 1 Day 1 may be used rather than repeating tests. All known sites of disease must be documented at screening and reassessed at each subsequent tumour evaluation. The same radiologic procedure used to assess disease sites at screening should be used throughout the

study. Response will be assessed by the investigator using RECIST v1.1 (refer to Section 17.3, Appendix 3).

Following disease progression, if a subject continues treatment beyond progression (contingent on satisfying criteria in section 5.8), and consent to ongoing treatment with single-agent osimertinib, patients will require ongoing 8-weekly CT scans until they come off trial. Subjects who do not satisfy criteria in section 5.8) will come off study, and will not require further imaging as a part of the trial.

6.2.5 Blood Collection

Local pathology laboratories will be used for routine blood tests. Blood will be taken prior to the administration of each cycle.

An **extra** blood draw for haematology and biochemistry (including liver function tests) as well as for cfDNA is required at day 15 of cycles 3 and 4.

6.2.6 EGFR-T790M mutation testing/ liquid biopsy

Prior to registration

Plasma testing confirming presence of EGFR sensitising mutation and T790M can be used to determine eligibility if tumour tissue sample is not obtainable prior to enrolment onto trial.

During the trial

Blood samples will be obtained for EGFR T790M biomarker evaluation from all eligible patients according to schedule 6.1, at day 1 (+/- 3 days) of each cycle, and on day 15 of cycles 3 and 4.

6.2.7 Tissue samples

Prior to registration

Provision of a tumour tissue sample is required at progression post first or second generation TKI from all patients prior to registration. The tissue specimen may be from either a primary or metastatic lesion, and tissue is obtained at the time of progression and prior to registration.

It would be preferable but not mandatory if tumour tissue was from a biopsy/resection within 28 days of enrolment onto the trial to provide the most up-to-date information regarding tumour and is microenvironment. The tumour tissue is used to test for presence of EGFR sensitising mutation and T790M for patient eligibility. Refer to Section 6.2.6 for alternative for test samples.

During the trial

A recommended but optional matched de novo tumour biopsy may also be obtained at disease progression. This biopsy may be performed on the primary tumour, a metastatic lesion. Alternatively, pleural effusion may be collected at this time if the participant has consented and processed to obtain a malignant pleural effusion cell pellet, which can be further processed into, and stored as a cytology block.

Follow up

Participants will be followed for at least 30 days after the last dose of study treatment as described in Section 8.2 for any new potentially related adverse events, or if adverse events are ongoing at the end-of-treatment visit.

Participants will be followed up until the final study endpoint is reached (i.e. death) by phone/clinic visit/medical record until the study is closed.

7 OUTCOMES, ENDPOINTS AND OTHER MEASUREMENTS

7.1 Progression free survival (PFS) at 12 months

PFS at 12 months is the primary endpoint for this trial. In this trial, 12 months is taken to mean 1 calendar year, i.e. 365 days, which corresponds to 13 four-weekly cycles plus 1 day. Study

participants who have stable disease, partial response or complete response confirmed at the first scheduled assessment after 12 months (week 56 CT scan) according to RECIST v1.1 will be considered to have attained PFS at 12 months.

PFS is measured from the date of registration until the date that disease progression is first observed, or the date of death from any cause, whichever occurs first. The date of first disease progression based on imaging is that of the first positive scan, even if this is determined in retrospect.

Participants who die without a reported prior progression will be considered to have progressed on the date of their death. Participants who did not have any study tumour assessments and did not die will be censored on the date they were registered (and considered non-evaluable for the purposes of PFS at 12 months). Participants who started any subsequent anti-cancer therapy without a prior reported progression will be considered to have progressed at that date.

7.2 Feasibility of alternating osimertinib and gefitinib

Feasibility for each patient is defined by whether they are able to complete 6 months of study treatment as per protocol without any dose interruption due to grade 3-5 adverse events.

7.3 Time to progression (TTP)

Time to progression measured (TTP) is the time from registration to objective tumour progression according to RECIST v1.1. Participants who die without evidence of progression are considered to have died from a competing risk.

7.4 Objective tumour response rate (OTRR = CR and/or PR)

The objective tumour response rate is defined as the proportion (percentage) of participants with a confirmed objective tumour response of either complete response (CR) or partial response (PR) assessed by the investigator according to RECIST v1.1 (refer to section 17.3, Appendix 3). For each participant, objective tumour response will be based on evaluable response assessments performed up until progression or commencement of non-protocol anti-cancer therapy, whichever occurs first.

A confirmed response is defined as a response (CR or PR) followed by confirmation of this response (CR or PR) at a subsequent assessment at least 4 weeks later.

7.5 Overall survival (OS)

OS time is defined as the interval from the date of registration to the date of death from any cause, or the date last followed up if known to be still alive.

7.6 Adverse events

The NCI Common Terminology Criteria for Adverse Events (NCI CTCAE) version 4.03 will be used to classify and grade the intensity of adverse events.

7.7 Tertiary/correlative measures

The feasibility of collection and processing of serial patient bloods for dynamic monitoring of EGFR T790M mutation will be evaluated with a view for clinical use in guiding targeted therapy.

Possible mechanisms of resistance in patients progressing on alternating osimertinib and gefitinib as identified in cfDNA or optional biopsy will be studied.

These may include changes in cfDNA levels of activating EGFR mutations including T790M and correlation with response as assessed by structural imaging. Quantification of changes in cfDNA levels of EGFR-T790M and classical activating mutations as analysed by using two main genomic technologies of Targeted amplicon-sequencing (Tam-Seq) and Droplet digital-PCR (ddPCR) in designated central laboratories. The presence of the EGFR T790M mutation at baseline and subsequent time points will be analysed by both frequencies and percentage. Changes of cfDNA

levels of mutations of interest will be correlated with response as assessed by tumour volume estimated from structural imaging, resistance mutation as identified by cfDNA or optional biopsy) and patterns of failure.

Other translational research studies may include identifying biomarkers that are prognostic and/or predictive of response to treatment, safety and resistance to study treatment (associations of biomarkers with clinical outcomes). Studies may include, but are not limited to:

- Studies that may help to understand the course of this cancer and related diseases;
- Biomarkers that may be RNA-based (single entity or entire expressed genome, RNA, miRNA), DNA-based (single entity or whole genome, germ line or tumour related), protein-based or other entities.

Since the identification of new biomarkers correlating with disease activity and the efficacy or safety of treatment is rapidly evolving, the definitive list of biomarkers remains to be determined.

8 SAFETY REPORTING

8.1 Definitions

An ADVERSE EVENT (AE) is any untoward medical occurrence in a patient or clinical investigational participant administered a pharmaceutical product and which does not necessarily have a causal relationship with this treatment. An AE can therefore be any unfavourable or unintended sign (including an abnormal laboratory finding), symptom, or disease temporally associated with the use of a medicinal investigational product, whether or not considered related to the medicinal product (see below).

AEs include the following:

- All suspected adverse drug or device reactions
- All reactions from drug or device – overdose, abuse, withdrawal, sensitivity, toxicity or failure of expected pharmacological action (if appropriate)
- Apparently unrelated illnesses
- Worsening (severity, frequency) of pre-existing illnesses or symptoms
- Injury or accidents
- Abnormalities in physiological testing or physical examination that require clinical intervention or further investigation (beyond ordering a repeat examination)
- Laboratory abnormalities that require clinical intervention or further investigation (beyond ordering a laboratory test). Any untoward event that occurs after the protocol-specified reporting period which the Investigator believes may be related to the drug or device.

A SERIOUS ADVERSE EVENT (SAE) is any untoward medical occurrence that at any dose:

- results in death,
- is life-threatening (i.e. the participant is at risk of death at the time of the event),
- requires inpatient hospitalisation or prolongation of existing hospitalisation,
- results in persistent or significant disability or incapacity,
- is a congenital anomaly/birth defect,
- other important medical events which, in the opinion of the investigator, are likely to become serious if untreated, or as defined in the protocol

NOTES:

(i) The term "life-threatening" in the definition of "serious" refers to an event in which the patient was at risk of death at the time of the event; it does not refer to an event which hypothetically might have caused death if it were more severe.

(ii) Important medical events which may not be immediately life-threatening or result in death or hospitalization but which may jeopardize the patient or may require intervention to prevent one of the listed outcomes in the definition above should also be considered serious.

A SUSPECTED UNEXPECTED SERIOUS ADVERSE REACTION (SUSAR) is an SAE that is related to the drug or device and is unexpected, i.e. not listed in the investigator brochure or approved Product Information; or is not listed at the specificity or severity that has been observed; or is not consistent with the risk information described in the Participant Information Sheet and Informed Consent Form or elsewhere in the protocol. An event is causally related if there is a reasonable possibility that the drug [intervention] caused the AE, i.e. there is evidence to suggest a causal relationship between the drug and the event.

For the purposes of this study, the following adverse events are not reported to the responsible coordinating centre as SAEs:

- Hospitalisations related to management of the disease under study
- Deaths related to disease under study
- Elective hospitalisations to facilitate the administration of treatment, e.g. Porta-Cath insertion
- Elective hospitalisations for other procedures, e.g. screening colonoscopy, stent change, cardiac catheter, etc.

8.2 Recording Adverse Events

AEs will be recorded from the first dose of study treatment until 30 days after cessation of study treatment. The National Cancer Institute Common Terminology Criteria for Adverse Events version 4 (NCI CTCAE v4.0) will be used to classify and grade the intensity of AEs after each treatment cycle. The worst grade will be recorded for each event.

The following information will be recorded for each SAE:

- Event description including classification according to NCI CTCAE
- Reason for classification as an SAE (death, hospitalisation etc.)
- Severity / worst grade
- Attribution to study intervention
- Expectedness (listed in IB/product information for osimertinib or gefitinib)
- Action taken with study intervention (for osimertinib or gefitinib)
- Outcome of SAE, including end date if recovered.

8.3 Pregnancy

In the event of a pregnancy occurring during the course of a study, the participant must be withdrawn from study drug immediately. Pregnancies occurring up to 6 months after the completion of the study drug must also be reported to the investigator. The investigator should counsel the patient; discuss the risks of continuing with the pregnancy and the possible effects on the foetus.

For chemotherapy and other studies where applicable: If the partner of a participant becomes pregnant while the participant is on treatment, then the investigator should counsel the patient; discuss the risks of continuing with the pregnancy and the possible effects on the foetus.

The NHMRC CTC must be notified within 24 hours and the participant followed during the entire course of the pregnancy and postpartum period. Parental and neonatal outcomes must be recorded even if they are completely normal. The NHMRC CTC will report pregnancies to AstraZeneca within 24 hours of being notified.

Pregnancy occurring in the partner of a participant and up to 30 days after the completion of the test drug should also be reported to the investigator and the NHMRC CTC. The partner should be counselled and followed as described above.

8.4 Reporting of Serious Adverse Events (including SUSARs)

The investigator is responsible for reporting all SAEs (including SUSARs) occurring during the study to the NHMRC CTC within 24 hours of investigational site staff becoming aware of the event according to the procedure documented in the Study Manual.

The NHMRC CTC will submit ‘reportable safety events’ to the TGA in Australia and to the lead site/coordinating centre in other regions, in time to comply with the requisite specified regulatory time windows (usually 7 days for fatal/life threatening events with an 8 day follow-up report, and 15 days for other SUSARs).

9 CENTRAL REVIEW

9.1 Imaging

Reports and images for tumour assessments will be kept locally at each site and should be retained in the event that central review is required.

10 CENTRAL STORAGE OF BIOSPECIMENS

10.1 Central Tissue Collection

Formalin-fixed paraffin-embedded (FFPE) tissue blocks or slides of tumour tissue will be collected for translational research from all participants at site and sent to the central tissue laboratories in Australia for central review (required of all participants) and translational studies (tertiary /correlative objectives) on these biospecimens and storage.

Refer to the Biological Sampling Manual for the details regarding central tissue collection.

10.2 Central Blood Collection

Central laboratories will be used to conduct translational studies including EGFR T790M mutation biomarker analyses. Blood will be collected and initially processed and stored at each site. Samples will later be shipped to a central facility for translational research and storage. Refer to the Biological Sampling Manual for procedures.

11 DRUG SUPPLY AND ACCOUNTABILITY

Investigational product	Dosage form and strength
**Osimertinib**	80mg Tablets<br>40mg Tablets
**Gefitinib**	250mg Tablets

11.1 Osimertinib

11.1.1 Formulation/ packaging/ storage

Osimertinib will be supplied by Astra Zeneca as tablets for oral administration as a single daily dose of 80mg. Each pack will contain 30 tablets of osimertinib 80mg.

Astra Zeneca will also supply packs of 30 tablets of osimertinib 40mg for use when dose modification is required for toxicity/adverse event management, see section 5.3.

Unopened packs of osimertinib tablets must be stored at approximately 25°C (77°F). Osimertinib must be used within the individually assigned expiry date on the label.

11.2 Gefitinib

11.2.1 Formulation/ packaging/ storage

Gefitinib will be supplied by Astra Zeneca as tablets for oral administration as a single daily dose of 250mg. Each pack will contain 30 tablets of gefitinib.

Unopened packs of gefitinib tablets must be stored at approximately 25°C (77°F). Gefitinib must be used within the individually assigned expiry date on the label.

11.3 Osimertinib and gefitinib supply

Osimertinib and gefitinib will be provided by Astra Zeneca for distribution by the sponsor.

11.4 Drug Accountability

The Pharmacy Department at participating institutions will maintain a record of drugs dispensed for each patient and subsequent returns. Patients will be asked to return unused drug and empty drug containers at each return visit.

The Pharmacy will also maintain a record of drug receipt and drug destruction.

12 STATISTICAL CONSIDERATIONS

12.1 Sample Size

A total sample size of 45 participants (allowing for 4 ineligible or non-evaluable) will distinguish the observed proportion alive and progression free at 12 months from 45% (not worthy of further research) versus 65% (worthy of further research) using a Simon's two-stage minimax design with 90% power with a 1-sided type 1 error rate of 10%.

The first 21 eligible and evaluable participants will comprise Stage 1 of the Simon minimax two-stage design. Stage 2 will comprise an additional 20 eligible and evaluable participants for a total of 41 eligible and evaluable participants. The null hypothesis will be rejected if there are 23 of the first 41 eligible and evaluable participants are alive and progression free at 12 months. The study will have 90% power to reject the null hypothesis if the true rate of PFS at 12-months is 65%. All evaluable participants consist of any registered participant.

12.2 Statistical Analysis

Analyses will include all participants registered into the study. The primary analysis of 12 month PFS will be based on the number and proportions of the first 41 eligible and evaluable participants who satisfy the criteria. The 12 month PFS rate will also be described with the Kaplan-Meier estimate and its 95% confidence interval (CI).

Progression free survival time, time to progression (TTP), and overall survival (OS) time will be summarised with Kaplan-Meier curves (or cumulative incidence curves for each competing risk type), and median times to each event with its 95% confidence intervals calculated using the method of Greenwood.

Analyses of safety outcomes will be restricted to patients who receive at least one dose of study treatment. Frequency and severity of adverse events (CTCAE v4.03) will be reported with associated 95% confidence intervals for the rates of serious grade (≥ 3). Worst overall grade during treatment will be computed and similarly reported.

OTRR and feasibility will be expressed as the proportions (percentages) of participants meeting the criteria along with suitable 95% confidence intervals.

Exploratory methods will be used to examine plasma cfDNA levels for EGFR and T790M mutations over time. The results will be described with graphical methods, including spider plots for individual participants, and simple summary statistics, for example the proportions of participants with increasing levels of T790M at the end of each 4 week cycle.

A detailed statistical analysis plan (SAP) will be prepared by the study statistician and approved by the TMC before starting the primary analyses.

12.3 Interim analyses

Consideration will be given to modifying the study protocol if:

- 9 or fewer of the first 21 evaluable participants are alive and progression free at 12 months.
- Clinically significant adverse events thought to be related to study treatment are unexpectedly frequent or severe.

13 STUDY ORGANISATION and COMMITTEES

13.1 Study coordination

The study is an investigator-initiated, academically-sponsored, cooperative group trial conducted under the auspices of ALTG in collaboration with the NHMRC CTC and University of Sydney. Coordination, monitoring, data acquisition and management and statistical analysis will be performed by the CTC.

13.2 Trial Management Committee

The Trial Management Committee (TMC) will oversee study planning, monitoring, progress, review of information from related research, and implementation of recommendations from other study committees and external bodies (e.g. ethics committees).

The TMC will consider whether to continue the study as planned, modify, or stop it, based on interim analyses or other information.

14 ADMINISTRATIVE ASPECTS

14.1 Ethics and regulatory compliance

In Australia, the study will be conducted according to the Note for Guidance on Good Clinical Practice (CPMP/ICH/135/95) annotated with TGA comments (Therapeutic Goods Administration DSEB July 2000) and in compliance with applicable laws and regulations. The study will be performed in accordance with the NHMRC Statement on Ethical Conduct in Research Involving Humans 2007, the NHMRC Australian Code for the Responsible Conduct of Research 2007, and the principles laid down by the World Medical Assembly in the Declaration of Helsinki 2008.

To this end, no patient will be recruited to the study until all the necessary approvals have been obtained and the patient has provided written informed consent. Further, the investigator shall comply with the protocol, except when a protocol deviation is required to eliminate immediate hazard to a participant. In this circumstance the NHMRC CTC, principal investigator and HREC must be advised immediately.

14.2 Confidentiality

The study will be conducted in accordance with applicable Privacy Acts and Regulations. All data generated in this study will remain confidential. All information will be stored securely at the

NHMRC CTC, University of Sydney and will only be available to people directly involved with the study and who have signed a Confidentiality Agreement.

14.3 Protocol amendments

Changes and amendments to the protocol can only be made by the Trial Management Committee. Approval of amendments by the Institutional HREC is required prior to their implementation. In some instances, an amendment may require a change to a consent form. The Investigator must receive approval/advice of the revised consent form prior to implementation of the change. In addition, changes to the data collected, if required, will be incorporated in the amendment.

The investigator should not implement any changes to, or deviations from, the protocol except where necessary to eliminate immediate hazard(s) to trial participant(s).

14.4 Data Handling and Record Keeping

All trial data required for the monitoring and analysis of the study will be recorded on the (e)CRFs provided. All required data entry fields must be completed. Data corrections will be done according to the instructions provided. The investigator will be asked to confirm the accuracy of completed CRFs by signing key CRFs as indicated.

Source documents pertaining to the trial must be maintained by investigational sites. Source documents may include a participant's medical records, hospital charts, clinic charts, the investigator's participant study files, as well as the results of diagnostic tests such as X-rays, laboratory tests, and electrocardiograms. The investigator's copy of the case report forms serves as part of the investigator's record of a participant's study-related data.

The following information should be entered into the participant's medical record:

a. The participant’s protocol identification.

b. The date that the participant entered the study, and participant number.

c. A statement that informed consent was obtained (including the date)

d. Relevant medical history

e. Dates of all participant visits and results of key trial parameters.

f. Occurrence and status of any SAEs

g. The date the participant exited the study, and a notation as to whether the participant completed the study or reason for discontinuation.

All study-related documentation at ANZ sites will be maintained for 15 years following completion of the study.

14.5 Study Monitoring

Data from this study will be monitored by Clinical Trials Program staff from the NHMRC CTC or their delegates. Monitoring will include centralised review of CRFs and other study documents for protocol compliance, data accuracy and completeness. Monitoring may include monitoring visits to investigational sites during the study for source data verification, review of the investigator’s site file and drug handling records. The NHMRC CTC will be given direct access to source documents, CRFs and other study-related documents. By signing the informed consent form, the participant gives authorised NHMRC CTC staff direct access to their medical records and the study data.

14.6 Audit and Inspection

This study may be participant to audit or inspection by representatives of the ALTG, Astra Zeneca (AZ) the CTC or representatives of regulatory bodies (e.g. Therapeutic Goods Administration (TGA)).

14.7 Clinical Study Report

A Clinical Study Report which summarises and interprets all the pertinent study data collected will be issued which may form the basis of a manuscript intended for publication. The Clinical Study Report or summary thereof will be provided to the study investigators, ALTG, AZ and the ethics committees. A lay summary of the results will be prepared for participants and other interested parties.

14.8 Publication Policy

The TMC may appoint a Writing Committee to draft manuscript(s) based on the trial data. Manuscript(s) will be submitted to peer-reviewed journal(s). The first publication will be the report of the full trial results based on the main protocol. The Writing Committee may develop a publication plan, including authorship, target journals and expected dates of publication. All publications must receive prior written approval from the TMC prior to submission.

15 **PROTOCOL AMENDMENTS**

Amendment no.	Date	Summary of change

16 REFERENCES

1. Jemal A BF, Center MM, Ferlay J, Ward E, Forman D. Global Cancer Statistics. Ca Cancer J Clin. 2011;61:69-90.
2. GLOBOCAN. Lung cancer- Estimated Incidence, Mortality and Prevalence Worldwide in 2012. 2012.
3. Molina JR YP, Cassivi SD, Schild SE, Adjei AA. Non-Small Cell Lung Cancer: Epidemiology, Risk Factors, Treatment and Survivorship. Mayo Clin Proc. 2008;83(5):584-94.
4. Kris MG JB, Berry LD, Kwiatkowski DJ, Iafrate AJ, Wistuba II, Varella-Garcia M et al. . Using Multiplexed Assays of Oncogenic Drivers in Lung Cancers to Select Targeted Drugs. JAMA. 2014;311(19):1998-2006.
5. Sequist LV SJ, Goldman JW, Wakelee HA, Gadgeel SM, Varga A, Papadimitrakopoulou V et al. . Rociletinib in EGFR-Mutated Non-Small Cell Lung Cancer. N Engl J Med. 2015;372:1700-9.
6. Janne PA YJ, Kim DW, Planchard D, Ohe Y, Ramalingam SS, Ahn MY et al. AZD9291 in EGFR Inhibitor-Resistant Non-small cell Lung Cancer. N Engl J Med. 2015;372(18):1689-99.
7. Lee CK, Brown C, Gralla RJ, Hirsh V, Thongprasert S, Tsai C-M, et al. Impact of EGFR Inhibitor in Non–Small Cell Lung Cancer on Progression-Free and Overall Survival: A Meta-Analysis. Journal of the National Cancer Institute. 2013.
8. Mok TS WY-L, Thongprasert S, Yang C-H, Chu D-T, Nagahiro S, Sunpaweravong P et al. Gefitinib or Carboplatin-Paclitaxel in Pulmonary Adenocarcinoma. N Engl J Med. 2009;361(10):947-57.
9. Mitsudomi T MS, Yatabe Y, Negoro S, Okamoto I, Tsurutani J, Seto T. Gefitinib versus cisplatin plus docetaxel in patients with non-small-cell lung cancer harbouring mutations of the epidermal growth factor receptor (WJTOG3405): an open label, randomised phase 3 trial. The Lancet Oncology. 2010;11:121-28.
10. Inoue A KK, Maemondo M, Sugawara S, Oizumi A, Isobe H, Gemma A et al. Updated overall survival results from a randomized phase III trial comparing gefitinib with carboplatin-paclitaxel for chemo-naive non-small cell lung cancer with sensitive EGFR gene mutations (NEJ002). Annals of oncology. 2013;24:54-9.
11. Rosell R CC, Gervais R, Vergnenegre A, Massuti B, Felip E, Palmero R. Erlotinib versus standard chemotherapy as first-line treatment for European patients with advanced EGFR mutation-positive non-small cell lung cancer (EURTAC): a mutlicentre, open-label, randomised phase 3 trial. The Lancet Oncology. 2012;13:239-46.
12. Zhou C, Wu YL, Chen G, Feng J, Liu XQ, Wang C, et al. Erlotinib versus chemotherapy as first-line treatment for patients with advanced EGFR mutation-positive non-small-cell lung cancer (OPTIMAL, CTONG-0802): a multicentre, open-label, randomised, phase 3 study. The Lancet Oncology. 2011;12(8):735-42.
13. Sequist LV, Yang JC-H, Yamamoto N, O'Byrne K, Hirsh V, Mok T, et al. Phase III Study of Afatinib or Cisplatin Plus Pemetrexed in Patients With Metastatic Lung Adenocarcinoma With EGFR Mutations. Journal of Clinical Oncology. 2013;31(27):3327-34.
14. Wu Y-L, Zhou C, Hu C-P, Feng J, Lu S, Huang Y, et al. Afatinib versus cisplatin plus gemcitabine for first-line treatment of Asian patients with advanced non-small-cell lung cancer harbouring EGFR mutations (LUX-Lung 6): an open-label, randomised phase 3 trial. The Lancet Oncology.15(2):213-22.
15. Engleman JA JP. Mechanisms of Acquired Resistance to Epidermal Growth Factor Receptor Tyrosine Kinase Inhibitors in Non-Small Cell Lung Cancer. Clini Cancer Res. 2008;14(2895-99).
16. Karlovich C GJ, Sun J-M, Mann E, Sequist LV, Konopa K, Wen W et al. . Assessment of EGFR mutation status in matched plasma and tumour tissue of NSCLC patients from a phase 1 study of rociletinib (CO-1686). Clinical Cancer Res. 2016;22(10):2386-95.
17. Piotrowska Z NM, Karlovich CA, Wakelee HA, Neal JW, Mino-Kenudson M, Fulton L et al. Heterogeneity Underlies the Emergence of EGFR T790M Wild-Type Clones Following Treatment of T790M-Positve Cancers with a Third Generation EGFR Inhibitor. Cancer Discov. 2015;5(7):713-22.

18. Chong CR JP. The quest to overcome resistance to EGFR-targeted therapies in cancer. Nat Med. 2013;19(11):1389-400.
19. Ohashi K MY, Michor F, Pao W. Epidermal Growth Factor Receptor Tyrosine Kinase Inhibitor-Resistant Disease. J Clin Oncol. 2013;31(8):1070-80.
20. Watanabe M KT, Isa S, Ando M, Tamiya A, Kubo A, Saka H et al. Ultra-Sensitive Detection of Pretreatment EGFR T790M Mutation in Non-Small Cell Lung Cancer Patients with an EGFR-Activating Mutation Using Droplet Digital PCR. Clini Cancer Res. 2015;21(15):3552-60.
21. Daniel CB KS. Whacking a mole-cule: clinical activity and mechanisms of resistance to third generation EGFR inhibitors in EGFR mutated lung cancers with EGFR-T790M. Transl Lung Cancer Res. 2015;4(6):809-15.
22. Sequist LV WB, Dias-Santagata D, Digumarthy S, Turke AB, Bergethon K, Shaw A et al. Genotypic and Histological Evolution of Lung Cancers Acquiring Resistance to EGFR Inhibitors. Sci Transl Med. 2011;3(75):1-27.
23. Niederst MJ SL, Poirier JT, Mermel CH, Lockerman EL, Garcia AR, Katayama R et al. RB loss in resistant EGFR mutant lung adenocarcinomas that transform to small-cell lung cancer. Nature Communications. 2015;6:1-10.
24. Engleman JA ZK, Mitsudomi T, Song Y, Hyland C, Park JO, Lindeman N. MET amplification leads to gefitinib resistance in lung cancer by activating ERBB3 signalling. Science. 2007;316(5827):1039-43.
25. Sequist LV YJ, Yamamoto N, O'Byrne K, Hirsh V, Mok T, Greater SL et al. Phase III Study of Afatinib or Cisplatin Plus Pemetrexed in Patients with Metastatic Lung Adenocarcinoma With EGFR Mutations. J Clin Oncol. 2013;31:3327-34.
26. Ramalingam SS BF, Krzakowski M, Barrios CH, Park K, Bover I, Heo DS et al. Randomized Phase II Study of Dacomitinib (PF-0029804), an Irreversible Pan-Human Epidermal Growth Factor Receptor Inhibitor, Versus Erlotinib in Patients with Advanced Non-Small Cell Lung Cancer. J Clin Oncol. 2012;30(27):3337-44.
27. Miller VA CJ, Chen YM, Park K, Kim SW, Zhou C, Wang M et al. . Afatinib versus placebo for patients with advanced, metastatic non-small cell lung cancer after failure of erlotinib, gefitinib, or both, and one of two lines of chemotherapy (LUX-Lung 1): a phase 2b/3 randomised trial. The Lancet Oncology. 2012;13:528-38.
28. Katakami N AS, Goto K, Hida T, Horai T, Inoue A, Ichinose Y et al. . LUX-Lung 4: A Phase II Trial Of Afatinib in Patients With Advanced Non-small Cell Lung Cancer Who Progressed During Prior Treatment with Erlotinib, Gefitinib, or Both. J Clin Oncol. 2013;31(27):3335-41.
29. Cross D AS, Ghiorghiu S, Eberlein C, Nebhan CA, Spitzler PJ, Orme JP et al. AZD9291, an irreversible EGFR TKI, overcomes T790M-mediated resistance to EGFR inhibitors in lung cancer. Cancer Discov 2014;4:9.
30. Janne PA BD, Camidge DR, Britten CD, Engelman JA, Garon EB, Guo F et al. . Phase I Dose-escalation Study of the Pan-HER Inhibitor, PF299804, in Patients with Advanced Malignant Solid Tumours. Clin Cancer Res. 2011;17(5):1131-39.
31. Goldberg SB OG, Digumarthy S, Muzikansky A, Jackman DM, Lennes IT, Sequist LV. Chemotherapy With Erlotinib or Chemotherapy Alone in Advanced Non-Small Cell Lung Cancer with Acquired Resistance to EGFR Tyrosine Kinase Inhibitors. The Oncologist. 2013;18:1214-20.
32. Soria JC WY, Nakagawa K, Kim SW, Yang JJ, Ahn MJ, Wang J et al. . Gefitinib plus chemotherapy versus placebo plus chemotherapy in EGFR-mutation-positive non-small-cell lung cancer after progression on first-line gefitinib (IMPRESS): a phase 3 randomised trial. The Lancet Oncology. 2015;16(8):990-8.
33. Mitsudomi T, Tsai C-M, Shepherd F, Bazhenova L, Lee JS, Chang G-C, et al., editors. AZD9291 in pre-treated T790M positive advanced NSCLC: AURA2 phase II study. Journal of Thoracic Oncology; 2015: ELSEVIER SCIENCE INC 360 PARK AVE SOUTH, NEW YORK, NY 10010-1710 USA.
34. Mok TS, Wu Y-L, Ahn M-J, Garassino MC, Kim HR, Ramalingam SS, et al. Osimertinib or Platinum–Pemetrexed in EGFR T790M–Positive Lung Cancer. New England Journal of Medicine. 2017; 376:629-640
35. Oxnard GR TK, Alden RS, Lawrance R, Paweletz CP, Cantarini M, Yang J CH et al. . Association between Plasma Genotyping and Outcomes of Treatment With Osimertinib in Advanced Non-Small-Cell Lung Cancer. J Clin Oncol. 2016;34:1-8.

36. Kim ES HV, Mok T, Socinski MA, Gervais R, Wu YL, Li LY et al. . Gefitinib versus Docetaxel in previously treated non-small-cell lung cancer (INTEREST): a randomised phase III trial. Lancet. 2008;372:1809-18.
37. Thress KS BR, Carr TH, Dearden S, Jenkins S, Brown H, Hammett T et al. . EGFR mutation detection in ctDNA from NSCLC patient plasma: A cross-platform comparison of leading technologies to support the clinical development of AZD9291. Lung Cancer. 2015;90:509-15.
38. Oxnard GR, Thress KS, Alden RS, Lawrance R, Paweletz CP, Cantarini M, et al. Association Between Plasma Genotyping and Outcomes of Treatment With Osimertinib in Advanced Non–Small-Cell Lung Cancer. Journal of Clinical Oncology. 2016;34(28):3375-82.
39. Thress KS PC, Felip E, Cho BC, Stetson D, Dougherty B, Lai Z et al. . Acquired EGFR C797S mutation mediates resistance to AZD9291 in non-small cell lung cancer harboring EGFR T790M. Nature Med. 2015:1-5.
40. Niederst MJ HH, Mulvey HE, Lockerman EL, Garcia AR, Piotrowska P, Sequist LV et al. . The allelic context of the C797S mutation acquired upon treatment with third generation inhibitors impacts sensitivity to subsequent treatment strategies. Clin Cancer Res. 2015;21(17):3924-33.

17 APPENDICES

17.1 Appendix 1 - Cockroft-Gault formula

Estimation and Measurement of Glomerular Filtration Rate (GFR)

Males:

$$\text{Creatinine CL (mL/min)} = \frac{\text{Weight (kg)} \times (140 - \text{Age})}{72 \times \text{serum creatinine (mg/dL)}}$$

Females:

$$\text{Creatinine CL (mL/min)} = \frac{\text{Weight (kg)} \times (140 - \text{Age})}{72 \times \text{serum creatinine (mg/dL)}} \times 0.85$$

Age = Age in years (20 to 80)
To convert serum creatinine in mg/dl to µmol/l use the following formula:
Cr (µmol/l) = Cr (mg/dl) x 88.4

17.2 *Appendix 2 – Guidance regarding potential interactions with concomitant medications*

Herbal and complementary therapies should not be encouraged because of unknown side effects and potential drug interactions. Concomitant medications are only recorded in the event of a serious adverse event report.

Osimertinib is an investigational drug for which limited data on in vivo interactions are currently available. Based on in vitro data and predicted clinical exposure data, osimertinib is considered unlikely to cause clinically significant drug interactions through inhibition or induction of cytochrome P450 enzyme activity. In vitro data have shown that the principal CYP enzymes responsible for the Phase I metabolism of osimertinib are CYP3A4, and CYP3A5.

1. **Drugs inhibiting CYP3A4 metabolism with a strong recommended against combining with osimertinib**

Based on small and clinically insignificant changes in AUC and C_{max} of osimertinib observed following co-administration with itraconazole, a potent CYP3A4 inhibitor, CYP3A4 inhibitors are not likely to affect the exposure of osimertinib. However, exposure to gefitinib was increased by about 1.8-fold (with a range increase from no increase to 3.3-fold). Based on the ability to increase exposure to gefitinib, potent CYP3A4 inhibitors should be ceased, if possible, 1 week prior to the commencement of treatment.

Table 2: Drugs inhibiting CYP3A4

Contraindicated drugs	Withdrawal period prior to osimertinib start
Clarithromycin, telithromycin, troleandomycin	1 week
Conivaptan	
Indinavir, lopinavir, nelfinavir, ritonavir, saquinavir, tipranavir, telaprevir, boceprevir, elvitegravir	
Itraconazole, ketoconazole, posaconazole, voriconazole	
Mibefradil	
Nefazodone	

This list is not intended to be exhaustive, and a similar restriction will apply to other agents that are known to strongly modulate CYP3A4 activity. Appropriate medical judgement is required.

2. **Drugs inducing CYP3A4 metabolism with a strong recommended against combining with osimertinib**

Based on the study of the effect of multiple oral doses of rifampicin, a potent CYP3A4 inducer, on the steady state exposure to osimertinib, a reduction in osimertinib $C_{ss,max}$ and AUC by approximately 73% and 78% respectively was observed. A single dose of rifampicin caused a reduction in gefitinib exposure of 60 to 90% within the individual.

We strongly recommend against concomitant use of strong CYP3A4 inducers with osimertinib. Based on the ability of strong CYP3A4 inducers to decrease osimertinib and gefitinib exposure, potent CYP3A4 inducers should be withdrawn, if possible, prior to study treatment.

Table 3: Drugs inducing CYP3A4

Contraindicated drugs	Withdrawal period prior to osimertinib start
Carbamazepine, phenobarbital, phenytoin, rifampicin, rifabutin, rifapentin<br>St John's Wort	3 weeks
Phenobarbitone	5 weeks

This list is not intended to be exhaustive, and a similar restriction will apply to other agents that are known to strongly modulate CYP3A4 activity. Appropriate medical judgement is required.

3. **Medicines whose exposures may be affected by osimertinib that may be allowed with caution**

Table 4: Exposure, pharmacological action and toxicity may be increased or decreased by osimertinib

Warning of possible interaction	Advice
Alfentanil<br>Amodiaquine<br>Repaglinide<br>Sirolimus<br>Tacrolimus<br>Torsemide<br>Fentanyl<br>Dihydroergotamine<br>Ergotamine<br>Quinidine<br>Carbemazepine<br>Simvastatin<br>Lovastatin<br>Atorvastatin<br>Rosuvastatin<br>Fluvastatin<br>Sulfasalazine<br>Warfarin<br>Phenytoin<br>S-Mephenytoin<br>Cyclosporine<br>Theophylline<br>Tizanidine<br>Aliskiren<br>Ambrisentan<br>Colchicine<br>Dabigatran<br>Digoxin<br>Fexofenadine<br>Maraviroc<br>Ranolazine<br>Talinolol<br>Tolyaptan	Drugs are permitted but caution should be exercised and patients monitored closely for possible drug interactions. Please refer to full prescribing information for all drugs prior to co-administration with osimertinib

There are currently no data confirming that there is a pharmacokinetic (PK) interaction between these agents and osimertinib; in vitro data suggest osimertinib has the potential to cause drug interactions at the intestinal level through CYP3A4 and BCRP, it has also been shown to be an

inhibitor of CYP2C8 and inducer of CYP3A4, CYP1A2 and CYP2C8 and p-glycoprotein. This list is not intended to be exhaustive, and a similar restriction will apply to other agents that are known to depend on CYP3A4, CYP2C8, CYP1A2 for metabolism or BCRP and p-glycoprotein for deposition disposition. Appropriate medical judgement is required.

4. Drugs that may prolong QT interval

The drugs listed in this section are taken from information provided by The Arizona Center for Education and Research on Therapeutics and The Critical Path Institute, Tucson, Arizona and Rockville, Maryland. Ref: http://www.arizonacert.org/medical-pros/drug-lists/drug-lists.htm.

4.1 Drugs known to prolong QT interval

Based on limited ECG parameter assessment during AURA1 and 2, the mean time-matched change from baseline in QTcF at Week 6 across all time points was 14.5ms (90% CI 14.0 to 15.0), with a maximum upper 90% CI limit on any time point being 17.5ms.
This magnitude of effect exerted by osimertinib is considered to be of limited clinical significance as evidenced by the low number of AEs reported under the QT prolongation SMQ in the Phase II studies. At the population level, no clinically significant changes in PR, RR and QRS intervals were identified during study treatment in any of the clinical trials.
The following drugs are known to prolong QT interval or induce Torsades de Pointes and are recommended to be withdrawn, where possible, prior to commencing osimertinib.

Table 5: Drugs prolonging QT interval

Contraindicated drug	Withdrawal period prior to osimertinib start
Clarithromycin, droperidol, erythromycin, procainamide	2 days
Cisapride, disopyramide, dofetilide, domperidone, ibutilide, quinidine, sotalol, sparfloxacin, thioridazine	7 days
Bepridil, chlorpromazine, halofantrine, haloperidol, mesoridazine	14 days
Levomethadyl, methadone, pimozide	4 weeks
Arsenic trioxide	6 weeks*
Amiodarone, chloroquine	1 year

* Estimated value as pharmacokinetics of arsenic trioxide has not been studied

4.2 Drugs that may possibly prolong QT interval

The use of the following drugs is permitted (notwithstanding other exclusions and restrictions) provided the patient has been stable on therapy for the periods indicated.

Table 6: Drugs that may prolong QT interval

Drug	Minimum treatment period on medication prior to osimertinib start
Alfuzosin, chloral hydrate, ciprofloxacin, dolasetron, foscarnet, galantamine, gemifloxacin, isradipine, ketoconazole, levofloxacin, mexiletine, nicardipine, octreotide, ofloxacin, ondansetron, quetiapine, ranolazine, telithromycin, tizanidine, vardenafil, venlafaxine, ziprasidone	2 days
Amantadine, amitriptyline, amoxapine, clozapine, doxepin, felbamate, flecainide, fluconazole, fosphenytoin, gatifloxacin, granisetron, imipramine, indapamide, lithium, moexipril/ HCTZ, moxifloxacin, risperidone, roxithromycin, sertraline, trimethoprim-sulfa, trimipramine, voriconazole	7 days
Azithromycin, citalopram, clomipramine, itraconazole,<br>nortriptyline, paroxetine, solifenacin, tacrolimus	14 days<br>5 weeks
Fluoxetine	
Protriptyline	6 weeks
Tamoxifen	8 weeks

17.3 Appendix 3 - RECIST v 1.1

Response Evaluation Criteria in Solid Tumours (RECIST v1.1)
These instructions are based on the guidelines recommended in Eisenhauer EA, Therasse P, Bogaerts J et al. New response evaluation criteria in solid tumours: Revised RECIST guideline (version 1.1). (Eur J Cancer, 2009; 45: 228-47)

Evaluable for response
All patients who have received at least one cycle of therapy and have their disease re-evaluated will be considered evaluable for response (exceptions will be those who exhibit objective disease progression prior to the end of cycle 1 who will also be considered evaluable). Patients on therapy for at least this period and who meet the other listed criteria will have their response classified according to the definitions set out below.

Disease and lesion definitions

Measurable Disease

Measurable *tumour lesions* are defined as those that can be accurately measured in at least one dimension (longest diameter to be recorded) as

- ≥ 20 mm with chest x-ray
- ≥10 mm with CT scan or clinical examination.
- Bone lesions are considered measurable only if assessed by CT scan and have an identifiable soft tissue component that meets these requirements (soft tissue component ≥ 10 mm by CT scan).

Malignant lymph nodes must be ≥ 15mm in the short axis to be considered measurable; only the short axis will be measured and followed.
All tumour measurements must be recorded in millimetres. Previously irradiated lesions are not considered measurable unless progression has been documented in the lesion.

Non-measurable Disease
All other lesions (or sites of disease), including small lesions are considered non-measurable disease. Bone lesions without a measurable soft tissue component, leptomeningeal disease, ascites, pleural/pericardial effusions, lymphangitis cutis/pulmonitis, inflammatory breast disease, lymphangitic involvement of lung or skin and abdominal masses followed by clinical examination are all non-measurable. Lesions in previously irradiated areas are non-measurable, unless progression has been demonstrated.

Target Lesions
When more than one measurable tumour lesion is present at baseline all lesions up to *a maximum of 5 lesions in total* (and a maximum of *2 lesions per organ*) representative of all involved organs should be identified as target lesions and will be recorded and measured at baseline. Target lesions should be selected on the basis of their size (lesions with the longest diameter), be representative of all involved organs, but in addition should be those that lend themselves to *reproducible repeated measurements*.
Note that pathological lymph nodes must meet the criterion of having a short axis of ≥ 15 mm by CT scan and only the *short* axis of these lymph nodes will contribute to the baseline sum. All other pathological lymph nodes (those with a short axis ≥ 10 mm but <15 mm) should be considered non-target lesions. Nodes that have a short axis < 10 mm are considered non-pathological and should not be recorded or followed. At baseline, the sum of the target lesions (longest diameter of tumour lesions plus short axis of target lymph nodes: overall maximum of 5) is to be recorded.
After baseline, a value should be provided on the CRF for all identified target lesions for each assessment, even if very small. If extremely small and faint lesions cannot be accurately measured but are deemed to be present, a default value of 5 mm may be used. If lesions are too small to measure and indeed are believed to be absent, a default value of 0 mm may be used.

Non-target Lesions

All non-measurable lesions (or sites of disease) plus any measurable lesions over and above those listed as target lesions are considered *non-target lesions*. Measurements are not required but these lesions should be noted at baseline and should be followed as “present” or “absent”.

Response Definitions

	**Evaluation**
Complete Response (CR)	Disappearance of all target and non-target lesions.<br>Any pathological lymph nodes (whether target or non-target) must have reduction in short axis to <10mm
Partial Response (PR)	At least a 30% decrease in the sum of the diameters of target lesions, taking as reference the baseline sum diameters. Non target lesions must be non-PD.
Stable Disease (SD)	Neither sufficient shrinkage to qualify for PR nor sufficient increase to qualify for PD, taking as reference the smallest sum diameter while on trial
Progressive Disease (PD)	At least a 20% increase in the sum of the diameters of target lesions, taking as reference the smallest sum on trial (this includes the baseline sum if that is the smallest on trial) AND an absolute increase of ≥ 5mm. Appearance of new lesions will also constitute progressive disease (including lesions in previously unassessed areas).<br>In exceptional circumstances, unequivocal progression of non-target disease may be accepted as evidence of disease progression, where the overall tumour burden has increased sufficiently to merit discontinuation of treatment or where the tumour burden appears to have increased by at least 73% in volume. Modest increases in the size of one or more non-target lesions are NOT considered unequivocal progression. If the evidence of PD is equivocal (target or non-target), treatment may continue until the next assessment, but if confirmed, the earlier date must be used.

Patients with measurable disease at baseline

Target lesions	Non-target lesions	New Lesions	Overall Response
CR	CR	No	CR
CR	Non-CR/Non-PD	No	PR
CR	Not all evaluated	No	PR
PR	Non-PD/ not all evaluated	No	PR
SD	Non-PD/ not all evaluated	No	SD
Not all evaluated	Non-PD	No	NE
PD	Any	Any	PD
Any	PD	Any	PD
Any	Any	Yes	PD

Patients with non-target disease only

Non-target lesions	New lesions	Overall response
CR	No	CR
Non-CR/Non-PD	No	Non-CR/Non-PD
Not all evaluated	No	NE
Unequivocal PD	Yes or No	PD
Any	Yes	PD

17.4 Appendix 4 - Common Terminology Criteria for Adverse Events (CTCAE)

Adverse events and/or adverse drug reactions will be recorded according to the Common Terminology Criteria for Adverse Events (CTCAE), version 4.03.

At the time this protocol was issued, the full CTC document was available on the NCI web site, at the following address:

http://evs.nci.nih.gov/ftp1/CTCAE/CTCAE_4.03_2010-06-14_QuickReference_8.5x11.pdf

17.5 Appendix 5 – ECOG Performance status scales

ECOG Performance Status Scale	
Grade	Descriptions
0	Normal activity. Fully active, able to carry on all pre-disease performance without restriction.
1	Symptoms, but ambulatory. Restricted in physically strenuous activity, but ambulatory and able to carry out work of a light or sedentary nature (e.g. light housework, office work).
2	In bed < 50% of the time. Ambulatory and capable of all self-care, but unable to carry out any work activities. Up and about more than 50% of waking hours.
3	In bed > 50% of the time. Capable of only limited self-care, confined to bed or chair more than 50% of waking hours.
4	100% bedridden. Completely disabled. Cannot carry on any self-care. Totally confined to bed or chair.
5	Dead.

As published in American Journal of Clinical Oncology:

Oken, M.M., Creech, R.H., Tormey, D.C., Horton, J., Davis, T.E., McFadden, E.T., Carbone, P.P.: Toxicity And Response Criteria Of The Eastern Cooperative Oncology Group. Am J Clin Oncol 5:649-655, 1982
