# Supplementary material for: A Phase II trial of alternating osimertinib and gefitinib therapy in advanced EGFR-T790M positive non-small cell lung cancer: OSCILLATE
Source: Nat Commun. 2024 Feb 28;15:1823. doi: 10.1038/s41467-024-46008-1 (PMC10902357; doi:10.1038/s41467-024-46008-1)
Supplement: Supplementary file 3 — Reporting Summary [file 41467_2024_46008_MOESM3_ESM.pdf]

nature portfolio

Corresponding author(s): Benjamin Solomon

Last updated by author(s): 2023-Dec-18

Reporting Summary

Nature Portfolio wishes to improve the reproducibility of the work that we publish. This form provides structure for consistency and transparency in reporting. For further information on Nature Portfolio policies, see our Editorial Policies and the Editorial Policy Checklist.

Statistics

For all statistical analyses, confirm that the following items are present in the figure legend, table legend, main text, or Methods section.

n/a	Confirmed	
☐	☒	The exact sample size (*n*) for each experimental group/condition, given as a discrete number and unit of measurement
☐	☒	A statement on whether measurements were taken from distinct samples or whether the same sample was measured repeatedly
☐	☒	The statistical test(s) used AND whether they are one- or two-sided<br>*Only common tests should be described solely by name; describe more complex techniques in the Methods section.*
☐	☒	A description of all covariates tested
☒	☐	A description of any assumptions or corrections, such as tests of normality and adjustment for multiple comparisons
☐	☒	A full description of the statistical parameters including central tendency (e.g. means) or other basic estimates (e.g. regression coefficient) AND variation (e.g. standard deviation) or associated estimates of uncertainty (e.g. confidence intervals)
☐	☒	For null hypothesis testing, the test statistic (e.g. *F*, *t*, *r*) with confidence intervals, effect sizes, degrees of freedom and *P* value noted<br>*Give P values as exact values whenever suitable.*
☒	☐	For Bayesian analysis, information on the choice of priors and Markov chain Monte Carlo settings
☒	☐	For hierarchical and complex designs, identification of the appropriate level for tests and full reporting of outcomes
☒	☐	Estimates of effect sizes (e.g. Cohen's *d*, Pearson's *r*), indicating how they were calculated

Our web collection on statistics for biologists contains articles on many of the points above.

Software and code

Policy information about availability of computer code

Data collection	Clinical data was recorder in electronic CRF (case report form) using InForm database. All entries in the eCRF had to be verifiable by source documents. There had to be a minimum documentation, which provided information on study participation and included all medical information necessary. Source documents had to mention that the subject had been included in an investigational study. There had to be no data that were inconsistent between the eCRF and source documents. All protocol-required information collected during the trial had to be entered by the investigator or designated representative into the eCRF. Patient data was documented pseudonymously. The investigator was responsible for ensuring that all sections of the eCRF was completed correctly. Any error had to be corrected in the eCRF and reason for change had to be entered. This had to be confirmed by dated electronic signature of the responsible investigator.
Data analysis	All analyses were performed using R version 3.6.3 and GraphPad PRISM version 9.1.2, and a two-sided p-value <0.05 was considered statistically significant. The Kaplan-Meier method was used to estimate PFS and OS. Prognostic impact of genomic data on PFS and OS was assessed using log-rank test. Cox proportional hazards regression was used to evaluate factors (exon 19 deletion, exon 21 L858R point mutation and sex) for association with PFS or OS. For biomarker analyses, group and sample comparisons were made using either two-tailed Mann-Whitney U test or Wilcoxon matched-pairs signed rank test, and Fisher's exact test was used to compare associations between categorical variables. All biomarker analyses were exploratory.

For manuscripts utilizing custom algorithms or software that are central to the research but not yet described in published literature, software must be made available to editors and reviewers. We strongly encourage code deposition in a community repository (e.g. GitHub). See the Nature Portfolio guidelines for submitting code & software for further information.

Data

Policy information about availability of data

All manuscripts must include a data availability statement. This statement should provide the following information, where applicable:

- Accession codes, unique identifiers, or web links for publicly available datasets
- A description of any restrictions on data availability
- For clinical datasets or third party data, please ensure that the statement adheres to our policy

The raw clinical data are protected and are not available due to data privacy laws. The de-identified datasets supporting the findings of this study are available for academic purposes on request from the corresponding authors, Professor Sarah-Jane Dawson (sarah-jane.dawson@petermac.org) and Professor Benjamin Solomon (ben.solomon@petermac.org), with the approval of the Institutional Ethics Committees. The trial protocol is available as a Supplementary Note in the Supplementary Information. The sequencing dataset generated in this study is deposited under the following accession number in the European Genome-phenome Archive (EGA) (https://ega-archive.org/studies): EGADxxx. (pending). All remaining data that support the findings of this study are available within the Article, the Supplementary Information or the Source Data file. Source data are provided in this paper.

Research involving human participants, their data, or biological material

Policy information about studies with human participants or human data. See also policy information about sex, gender (identity/presentation), and sexual orientation and race, ethnicity and racism.

Reporting on sex and gender	Findings does not apply to only one sex or gender and although sex and gender were not considered in the study design, an unplanned analysis of the primary endpoint by sex was performed. Sex and/or gender was determined based on self-reporting. Disaggregated sex data was collected (female: 29, male:18). Sex based analyses were performed and reported in manuscript.
Reporting on race, ethnicity, or other socially relevant groupings	Findings does not apply to one race or ethnicity and race and ethnicity were not considered in the study design. Race and other socially relevant ethnicity were provided by the participants and classification into different categories were based on self-reporting. No subgroup analyses regarding race, ethnicity or other socially relevant groupings were performed
Population characteristics	Adults with advanced, EGFR mutated NSCLC with acquired T790M mutation and resistance to first or second generation EGFR tyrosine kinase inhibitors (TKIs), with a median age of 60 years old (range 32-86). 29 female and 18 male participants were included. 66% of participants were never smokers and 32% were former smokers. 60% of participants were non-Asian and the median number of prior systemic therapies was 1 (range 1-2). The most common activating EGFR mutation based on tumor genotyping was exon 19 deletion (64%), followed by exon 21 L858R (34%) and all patients had EGFR T790M confirmed through tumor (45%) and/or ctDNA analysis (55%), based on local laboratory testing at each participating site.
Recruitment	Participants were identified and recruited by medical oncologists from 12 tertiary hospitals around Australia. There are no selection bias identified. The details of the inclusion criteria of participants are shown in the study protocol. This is a phase II, single arm clinical trial to assess efficacy and safety of alternating osimertinib and gefitinib in patients with advanced, EGFR T790M positive NSCLC. Serial ctDNA analyses were performed to assess therapeutic response, understand clonal dynamics, and characterize genomic mechanisms of resistance to alternating therapy. Informed consent was obtained from all human research subjects.
Ethics oversight	This study was approved by Sydney Local Health District Ethics Review Committee (RPAH Zone)

Note that full information on the approval of the study protocol must also be provided in the manuscript.

Field-specific reporting

Please select the one below that is the best fit for your research. If you are not sure, read the appropriate sections before making your selection.

☒ Life sciences ☐ Behavioural & social sciences ☐ Ecological, evolutionary & environmental sciences

For a reference copy of the document with all sections, see nature.com/documents/nr-reporting-summary-flat.pdf

Life sciences study design

All studies must disclose on these points even when the disclosure is negative.

Sample size	A total of 45 participants provides 90% power, with a 1-sided type 1 error rate of 10%, to distinguish the observed proportion alive and progression free at 12 months from true rates of 45% (not worthy of pursuit) and 65% (worthy of pursuit) using a Simon, 2-stage, minimax design allowing for 4 ineligible or in-evaluable participants.
Data exclusions	Previous or current treatment with osimertinib, or other drugs that target EGFR-T790M mutations, symptomatic CNS disease, known interstitial lung disease or any unstable systemic disease were ineligible for this study.
Replication	Replication of clinical trial data is not feasible. Targeted sequencing and ddPCR were not applicable for replication due to limitation of plasma samples.

Randomization	This was a single-arm, non-randomized clinical trial
Blinding	Blinding is not relevant as this was a single arm study and all participants received study drug

Reporting for specific materials, systems and methods

We require information from authors about some types of materials, experimental systems and methods used in many studies. Here, indicate whether each material, system or method listed is relevant to your study. If you are not sure if a list item applies to your research, read the appropriate section before selecting a response.

Materials & experimental systems

n/a	Involved in the study
☒	☐ Antibodies
☒	☐ Eukaryotic cell lines
☒	☐ Palaeontology and archaeology
☒	☐ Animals and other organisms
☐	☒ Clinical data
☒	☐ Dual use research of concern
☒	☐ Plants

Methods

n/a	Involved in the study
☒	☐ ChIP-seq
☒	☐ Flow cytometry
☒	☐ MRI-based neuroimaging

Clinical data

Policy information about clinical studies

All manuscripts should comply with the ICMJE guidelines for publication of clinical research and a completed CONSORT checklist must be included with all submissions.

Clinical trial registration	Clinical trial registration ACTRN12617000720314
Study protocol	Full protocol provided in Supplementary information
Data collection	Participants were recruited from 12 tertiary centres in Australia, from September 4, 2017 to June 11, 2019 and data was collected when patient presented for their clinical visit during this time period. Clinical data was recorded in the electronic Case Report Form (eCRF) using the InForm database. All entries in the eCRF had to be verifiable by source documents. There had to be a minimum documentation, which provided information on study participation and included all medical information necessary. Source documents had to mention that the subject had been included in an investigational study. There had to be no data that were inconsistent between the eCRF and source documents. All protocol-required information collected during the trial had to be entered by the investigator or designated representative into the eCRF. Patient data was documented pseudonymously. The investigator was responsible for ensuring that all sections of the eCRF was completed correctly. Any error had to be corrected in the eCRF and reason for change had to be entered. This had to be confirmed by dated electronic signature of the responsible investigator.
Outcomes	Primary outcome was defined as progression free survival at 12 months according to RECIST vl.l. and secondary outcomes were feasibility of alternating regimen, objective tumor response rate as per RECIST vl.1, overall survival and frequency and severity of adverse events as defined by CTCAE v4.03

Plants

Seed stocks	NA
Novel plant genotypes	NA
Authentication	NA
