# Supplementary material for: A Phase II trial of alternating osimertinib and gefitinib therapy in advanced EGFR-T790M positive non-small cell lung cancer: OSCILLATE
Source: Nat Commun. 2024 Feb 28;15:1823. doi: 10.1038/s41467-024-46008-1 (PMC10902357; doi:10.1038/s41467-024-46008-1)
Supplement: Supplementary file 4 — Description of Additional Supplementary Files [file 41467_2024_46008_MOESM4_ESM.pdf]

Description of Additional Supplementary Files

File Name: Supplementary Data 1
Description: ctDNA mutation data for all analyzed samples. ctDNA, circulating tumor DNA.

File Name: Supplementary Data 2
Description: List of genes from AVENIO ctDNA panel
